# RNA interference of *Aspergillus flavus* in response to Aspergillus flavus partitivirus 1 infection

**DOI:** 10.3389/fmicb.2023.1252294

**Published:** 2023-11-14

**Authors:** Yinhui Jiang, Xiang Liu, Xun Tian, Jianhong Zhou, Qinrong Wang, Bi Wang, Wenfeng Yu, Yanping Jiang, Tom Hsiang, Xiaolan Qi

**Affiliations:** ^1^Key Laboratory of Endemic and Ethnic Diseases, Ministry of Education, Guizhou Medical University, Guiyang, China; ^2^Key Laboratory of Medical Molecular Biology of Guizhou Province, Guizhou Medical University, Guiyang, China; ^3^Department of Dermatology, The Affiliated Hospital, Guizhou Medical University, Guiyang, China; ^4^School of Environmental Sciences, University of Guelph, Guelph, ON, Canada

**Keywords:** *Aspergillus flavus*, mycoviruses, RNA-dependent RNA polymerase, dicer, argonaute, antiviral response, small RNA

## Abstract

RNA interference (RNAi) is one of the important defense responses against viral infection, but its mechanism and impact remain unclear in mycovirus infections. In our study, reverse genetics and virus-derived small RNA sequencing were used to show the antiviral responses of RNAi components in *Aspergillus flavus* infected with Aspergillus flavus partitivirus 1 (AfPV1). qRT-PCR revealed that AfPV1 infection induced the expression of the RNAi components in *A. flavus* compared with noninfected *A. flavus*. Knock mutants of each RNAi component were generated, but the mutants did not exhibit any obvious phenotypic changes compared with the *A. flavus* parental strain. However, after AfPV1 inoculation, production of AfPV1 was significantly less than in the parental strain. Furthermore, sporulation was greater in each AfPV1-infected mutant compared with the AfPV1-infected parental *A. flavus*. We also investigated the sensitivity of virus-free and AfPV1-infected RNAi mutants and the parental strain to cell wall stress, osmotic stress, genotoxic stress, and oxidative stress. The mutants of DCLs and AGOs infected by AfPV1 displayed more changes than RDRP mutants in response to the first three stresses. Small RNA sequencing analysis suggested that AfPV1 infection reduced the number of unique reads of sRNA in *A. flavus*, although there were many vsiRNA derived from the AfPV1 genome. GO term and KEGG pathway analyses revealed that the functions of sRNA affected by AfPV1 infection were closely related to vacuole production. These results provide a better understanding of the functional role of RNAi in the impact of AfPV1 on the hypovirulence of *A. flavus*.

## Introduction

Mycoviruses are viruses that infect fungi, mostly asymptomatically and frequently with mixed infections in a host (Ghabrial et al., 2015; Sutela et al., 2019). Some mycoviruses do cause obvious phenotypic changes in their host (Xiao et al., 2014). Because mycoviruses could cause hypovirulence in their fungal hosts, researchers began to focus on the possibility of using mycoviruses to control or manage fungal pathogens of plants, insects, or humans (Kotta-Loizou, 2021). Moreover, with improvements in high-throughput sequencing, an increasing number of novel mycoviruses are being identified, implying huge potential advances in this field (Raco et al., 2022). The genomes of most mycoviruses are composed of double strands (ds) RNA and encode RNA-dependent RNA polymerases (RDRPs) (Ahlquist, 2002).

RNAi is regarded as one of the antiviral response mechanisms in fungi (Chun et al., 2020). RNAi is conserved among eukaryotes, is a gene-silencing mechanism, and regulates gene expression through the recession of messenger RNA (mRNA) triggered by the interference of dsRNA molecules (Meister and Tuschl, 2004). The regular RNAi pathway contains a series of organism cellular proteins, which include DCL-like (DCL) proteins, argonaute-like (AGO) proteins, and RDRP proteins (Ahlquist, 2002). Specificity of the defense is afforded by small interfering RNAs (siRNAs, 21–24 nt in length) generated from dsRNA molecules, including viral dsRNAs by DCL proteins that are RNase III-type enzymes (Baulcombe, 2004). siRNA binds with AGO proteins to form RNA-induced silencing complexes (RISC), and the RISC directly degrades the complementary sequences (Ahlquist, 2002). The RDRP proteins are responsible for the conversion of single-stranded (ss) RNA into dsRNA molecules, and hence amplify siRNA signals (Ahlquist, 2002). The functions of DCL and AGO proteins have been deeply researched in plants and animals. In plants, microRNAs (miRNAs, 21–24 nt in length) are generated by DCL proteins (Blevins et al., 2006). AGO proteins carry miRNAs with a bias for 5′-end nucleotides, AGO2 and AGO4 preferentially recruit sRNAs with adenosine (A) at 5′-end, and AGO1 favors 5′-end with uridine (U) (Xie et al., 2004; Carbonell, 2017; Meyers and Axtell, 2019). However, in fungi, the functions of these proteins and their involvement in RNAi are less well studied. In fungi, RNAi was first found and described in *Neurospora crassa* (Cogoni and Macino, 1999). Similar to plants and animals, the DCL, AGO, and RDRP proteins are also three core enzymes for small RNA (sRNA) produced in fungi, and different size categories of sRNAs in fungi are produced by different DCL and AGO proteins (Axtell, 2013; Nguyen et al., 2018).

Several studies have found RNAi-mediated antiviral defense mechanisms in fungi such as *Cryphonectria parasitica*, *Aspergillus nidulans*, *Rosellinia necatrix*, *Fusarium graminearum*, and *Pyricularia orizae* (Segers et al., 2007; Hammond et al., 2008; Yaegashi et al., 2016; Nguyen et al., 2018; Yu et al., 2018). The RNAi-mediated antiviral defense of *C. parasitica* against *Cryphonectria parasitica* hypovirus 1 (CHV1) has been well studied, and it involves the induction of *dcl2* and *ago2* transcripts and the production of hairpin RNA (Segers et al., 2007; Xuemin et al., 2008; Qihong et al., 2009). Moreover, CHV1 encodes a p29 protein regarded as a suppressor in *C. parasitica*, which inhibits the gene expression induced by RNAi-mediated viral defense (Segers et al., 2006; Xuemin et al., 2008). Additionally, genes associated with RNAi of *R. necatrix* and *F. graminearum* were differentially induced by infection from distinct dsRNA mycoviruses (Chiba et al., 2013; Yu et al., 2018). For example, infection with Rosellinia necatrix mycovirus 3 (RnMyRv3) or Rosellinia necatrix megabirnavirus 1 (RnMBV1) upregulates the expression of genes DCL2, AGO2, RDRP1, and RDRP2 in *R. necatrix* (Yaegashi et al., 2016). The transcripts of *dcl2* and *ago1* of *F. graminearum* accumulate at lower levels after infection by Fusarium graminearum virus 1 (FgV1) than by FgV2 or FgV3, and AGO1 also involves the accumulation of virus-derived siRNAs (vsiRNAs) in FgV1-infected *F. graminearum* strain (Yu et al., 2018).

*Aspergillus flavus* is regarded as an opportunistic pathogen, which causes aspergillosis diseases in immunocompromised individuals (Rudramurthy et al., 2019). Moreover, *A. flavus* produces aflatoxin, a hepatocarcinogenic secondary metabolite (Klich, 2007). Currently, only antifungal antibiotics are used to treat aspergillosis (Arendrup, 2014; Paul et al., 2015). However, mycovirus-mediated hypovirulence could be used as a new type of therapy for human pathogenic fungi (Kotta-Loizou and Coutts, 2017; van de Sande and Vonk, 2019). In the previous studies, our research group found that AfPV1 is placed as a new genus in *Partitiviridae* family, and AfPV1-infected *A. flavus* showed debilitation and hypovirulence in animal and insect studies (Jiang et al., 2019, 2022). However, the interactions between the AfPV1 and *A. flavus* are not well understood. *A. flavus* RNAi components contain three DCLs (DCL1, DCL2, and DCL3), three AGOs (AGO1, AGO2, and AGO3), and three RDRPs (RDRP1, RDRP2, and RDRP3) (Nakayashiki and Nguyen, 2008). In this study, we constructed RNAi component mutants for each of DCL1, DCL2, AGO1, AGO2, RDRP1, RDRP2, and RDRP3, and analyzed sRNA profiles in *A. flavus* to explore the role of each RNAi component in AfPV1 infection of *A. flavus*.

## Materials and methods

### Fungal strains and growth conditions

The virus-free *A. flavus* strain LD-F1 carrying a pyrithiamine resistance (*ptr*) gene, and the AfPV1-infected strain LD-F1-b were described previously (Jiang et al., 2019). The *A. flavus* parental strain CA14 (genotype: *Δku70*, *ΔpyrG::pyrG*) was provided by Professor Shihua Wang (School of Life Sciences, Fujian Agriculture and Forestry University, Fuzhou, Fujian, China), and has been described previously (Yang et al., 2019). All *A. flavus* strains were maintained on potato dextrose agar (PDA) at 30°C for mycelial growth and conidiation assays.

### Sequence analysis of RNAi proteins

The protein sequences of the RNAi components were downloaded from the National Center for Biotechnology Information database (NCBI). The conserved domains of RNAi proteins were predicted using online software (Domains & Structure^1^) in NCBI, and the visualized domains were constructed with TBtools (Chen et al., 2020).

### Generation of gene deletion mutants and virus transfection

The DNA constructs for gene deletion were obtained using the modified double joint (DJ) PCR method (Yu et al., 2004). Briefly, to generate the DCL-1 gene deletion mutant, the 5′ and 3′ flanking regions of the gene were amplified from *A. flavus* isolate CA14 using the primer pairs DCL1-1F/−1R and DCL1-2F/−2R, respectively. A pyrithiamine resistance gene (*ptr*) used as a selectable marker, was amplified from vector pPTRI (Takara, Dalian, China) using primer pair Ptr-F/Ptr-R (Chang et al., 2010). The three amplicons were mixed with a 1:3:1 molar ratio and fused with the DJ PCR method as previously described (Yu et al., 2004). This strategy was also used to generate the DCL2, AGO1, AGO2, RDRP1, RDRP2, and RDRP3 gene-deletion mutants. The final DNA disruption constructs were transformed into *A. flavus* isolate CA14 by using a PEG-mediated method with slight modifications (Chang et al., 2010). All transgenic isolates were identified by PCR and Southern blot hybridization. The fungal virus AfPV1 was introduced into CA14, and each transformant strain through hyphal anastomosis from AfPV1-infected strain LD-F1-b (Supplementary Figure S1). After more than five generations of screening on 2 mg/mL 5-fluorowhey acid (which does not permit LD-F1-b to grow), the positive infections were confirmed by RT-PCR using virus-specific primer pairs dsRNA2-F/R (Supplementary Figure S1). All the primer sequences are shown in Supplementary Table S1.

### DNA extraction and southern blot hybridization

Fungal isolates were grown on sterilized cellophane films placed on PDA at 30°C in the dark for 5 days. Genomic DNA from the mycelia of each isolate was extracted using CTAB (Cetyltrimethylammonium Bromide) as previously described (Posadas et al., 2012). For the Southern blot hybridization experiment, approximately 10 μg of genomic DNA was digested by the appropriate restriction enzyme. After digestion, DNA was loaded on a 1% agarose gel and electrophoresed at 30 V for 10 h and 4°C. Then the gels were soaked in 50 mM NaOH for 30 min at room temperature, and neutralized with 20× SSC (3.0 M NaCl, 0.3 M sodium citrate, pH 7.0) for 45 min. The digested DNA was transferred to a nylon membrane (GE Healthcare, Buckinghamshire, United Kingdom) by capillary action with 20× SSC for 24 h and cross-linked to the membrane by UV irradiation (UVP CX-2000, USA). Hybridization and detection were performed using a DIG High Prime DNA Labeling and Detection Starter Kit II (Roche, Mannheim, Germany). The probe sequences are shown in Supplementary material 1.

### Analyses of growth, conidia, and sclerotia

Conidia (1 × 10^6^ spores/mL, 3 μL) from each *A. flavus* strain were placed onto the center of PDA plates (9 cm in diameter), and cultured at 30°C in the dark. The radial growth rate (RGR) was calculated for each developing colony (five replicates for each fungal isolate) at 2-day intervals over a week as follows: RGR (cm/d) = [(D 4 − D 2)/2]/2, where D 4 and D 2 represent the diameter of 4- and 2-day-old colonies, respectively.

Sporulation was assessed for cultures incubated on PDA plates at 30°C in the dark for 6 days (five replicate plates for each strain). The conidia were washed off with 2 mL saline buffer, and spores were counted using a hemocytometer.

To observe sclerotia, conidia (1 × 10^6^ spores/mL, 3 μL) of each *A. flavus* strain were placed onto Wickerham agar medium and incubated at 37°C in darkness for 7 days following Raper and Thom (1949). The experiment was done with five replicate cultures for each isolate and repeated three times.

### Stress assay

Measurements of *A. flavus* stress tolerance were done with slight modifications following previous methods (Takahashi-Nakaguchi et al., 2019, 2020; Sun et al., 2021). Conidia (1 × 10^6^ spores/mL, 3 μL) of each *A. flavus* strain were placed onto a yeast-glucose minimal medium (YGM: 0.1% yeast extract powder, 1% glucose, and 1.5% agar) supplemented with the following agents separately: cell wall stress agent (Congo red, CR); hyperosmotic stress mediator sodium chloride (NaCl), genotoxic stress agent methyl methanesulfonate (MMS); or oxidative stress agent hydrogen peroxide (H_2_O_2_). The cultures of each plate were incubated at 30°C in darkness for 5 days. Colony diameters were measured every day (five replicate plates for each strain and repeated three times). The percent radial growth inhibition (RGI) was determined as follows: (radial colony diameters without any stress – radial colony diameters with stress)/radial colony diameters without any stress × 100.

### RNA extraction and real-time reverse transcription-quantitative PCR (qRT-PCR)

The mycelia of *A. flavus* strains were placed onto sterilized cellophane films placed on PDA, and cultured at 30°C in the dark for 5 days. Total RNA was extracted from the mycelia using the TRIzol reagent (Invitrogen, Grand Island, Germany). To detect the relative expression levels of RNAi-related genes, including DCL-1, DCL-2, AGO-1, AGO-2, RDRP-1, RDRP-2, and RDRP-3, 2 μg of total RNA were denatured at 65°C for 10 min and chilled on ice. The denatured RNA was used for reverse transcription (RT) reaction using PrimeScript™ RT reagent Kit with gDNA Eraser (Takara, Dalian, China). TB Green® *Premix Ex Taq*™ (Takara, Dalian, China) was used for qPCR to detect the expression of the RNAi-related genes. For accumulation of AfPV1 in *A. flavus*, total RNA or dsRNA was denatured at 95°C for 15 min and chilled on ice, and then subjected to qRT-PCR as described above. The primers used for RT-PCR and qPCR analyses are listed in Supplementary Table S1. Two replicates for each of the three RNA samples from independent experiments were analyzed.

### Analysis and sequencing of sRNA

Total RNA from fungal cultures were prepared as described above. The RNA samples were processed using the small-RNA sequencing service by Novogene, Ltd. (Beijing, China) on the Illumina HiSeq 2000 platform. Low-quality data and adapters were removed before analysis. The sequencing reads were mapped to a reference genome sequence (GCF_014117465.1) for removing contamination or mis-sequencing. The non-coding RNAs (ncRNAs) were identified by comparison to the Rfam V13.0 database^2^, and microRNAs (miRNAs) were identified using miRBase V22^3^. Based on the taxonomic identity of the species, we obtained the ncRNA sequence of the species from Rfam and miRBase, and then mapped the reads to these sequences, which were then considered known non-coding RNAs. However, sRNAs with no information in miRBase and Rfam directly were considered putative miRNAs using MIREAP^4^. All sRNAs were also mapped to each genome segment (GenBank sequences MK344768, MK344769, and MK344770) of AfPV1 to identify the vsiRNAs. The targets of vsiRNA were predicted using TargetScan^5^. The siRNA sequencing raw data have been submitted to the National Center for Biotechnology Information website^6^ under project No. PRJNA954166. All targets were clustered into Gene Ontology (GO) terms using the online database^7^. KEGG (Encyclopedia of Genes and Genomes^8^) pathway analysis was also performed to identify target-enriched pathways. The *p-*value ≤0.05 was used for calculations of GO and KEGG significance.

## Results

### Identification of homologous RNAi proteins in *Aspergillus flavus*

**Figure 1:**
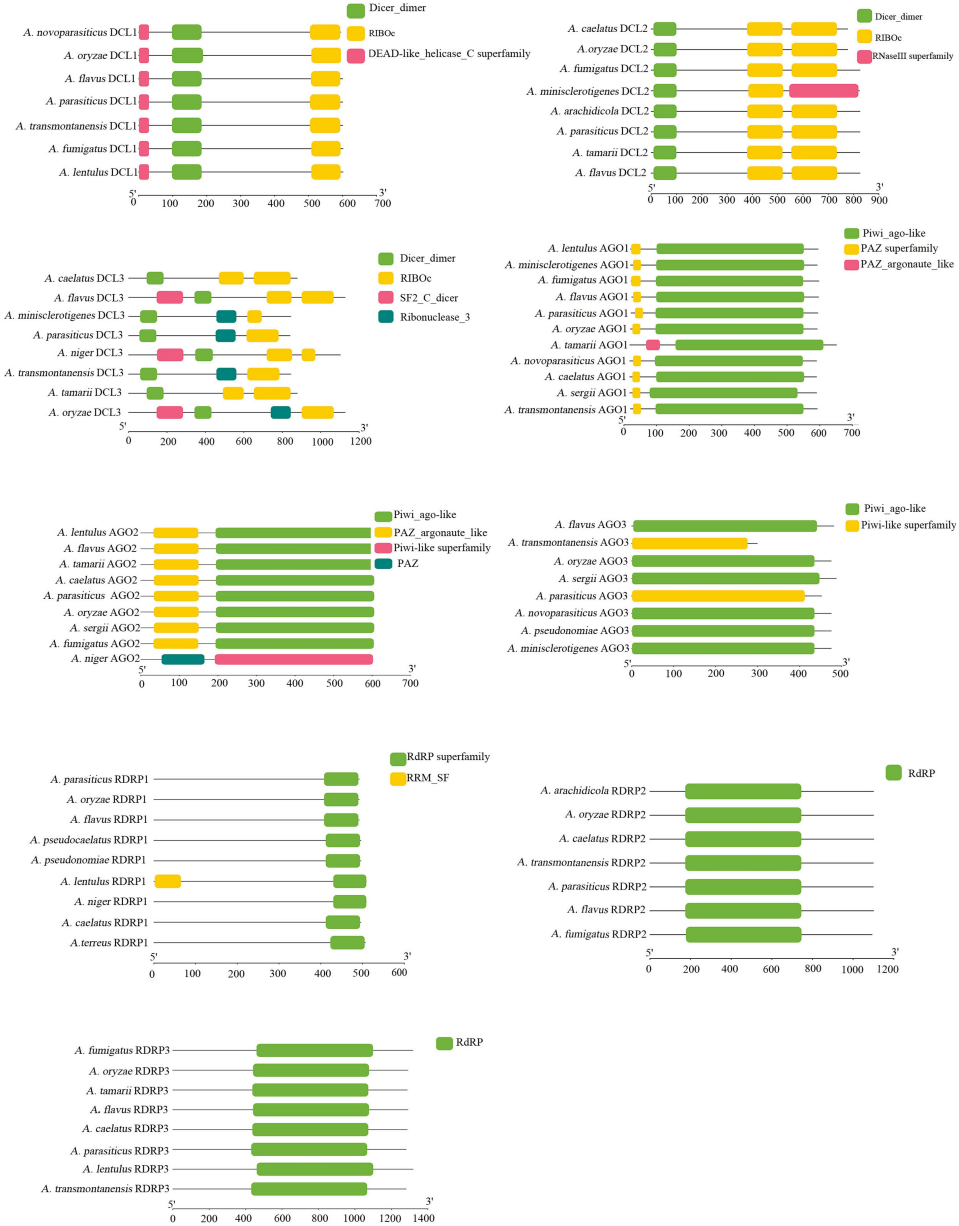
Bioinformatic analysis of RNAi protein domain analysis of *A. flavus*, *A. oryzae*, *A. transmontanensis*, *A. minisclerotigenes*, *A. parasiticus*, *A. arachidicola*, *A. pseudonomiae*, *A. sergii*, *A. tamarii*, *A. pseudocaelatus*, *A. caelatus*, *A. lentulus*, *A. niger*, *A. fumigatus*, *A. thaliana*. The accession numbers of RNAi proteins are shown in Supplementary Table S2.

The sequences of homologous proteins for RNAi components in *A. flavus* were appraised by searching the NCBI database, and the accession numbers are shown in Figure 1. There were three DCL proteins (DCL1, DCL2, and DCL3), three AGO proteins (AGO1, AGO2, and AGO3), and three RDRP proteins (RDRP1, RDRP2, and RDRP3) found in *A. flavus*. The lengths of the amino acid sequence above were similar, and they often revealed the same protein domain with highly conserved sequences (Figure 1). However, the RNaseIII superfamily domain is unique in DCL2 of *A. minisclerotigenes*, the PAZ_argonaute_like domain is unique in AGO1 of *A. tamarii*, and the Piwi-like superfamily and the PAZ domain is unique in AGO2 of *A. niger* (Figure 1).

### RNAi genes of *Aspergillus flavus* in response to AfPV1 infection

**Figure 2:**
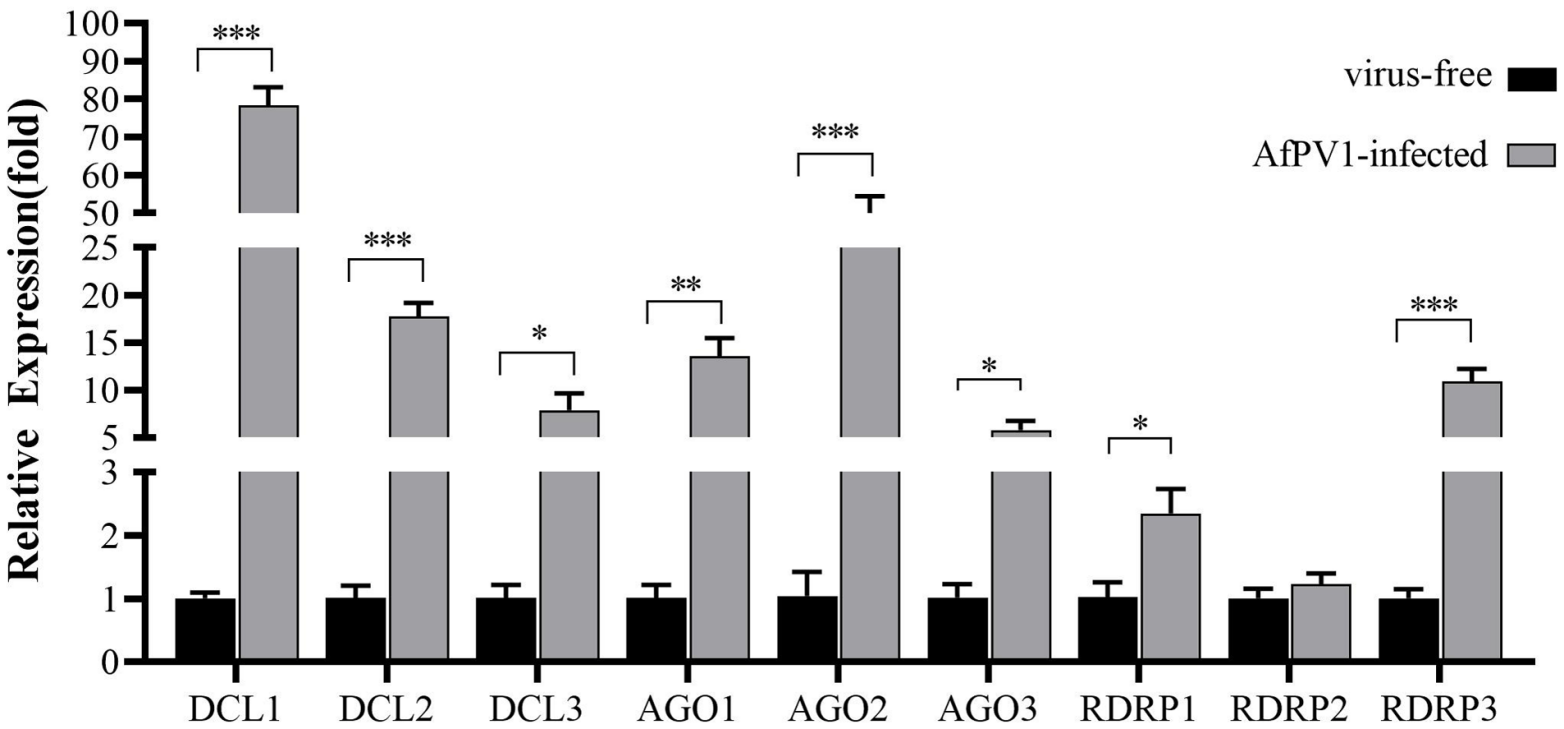
Transcript accumulation of RNAi components in response to infection by AfPV1 in *A. flavus*. Significant differences between *A. flavus* virus-free strain CA14 and AfPV1-infected CA14 strains are shown with ∗ (*p* < 0.05), ∗∗ (*p* < 0.01), ∗∗∗ (*p* < 0.001), by Dunnett’s test.

The transcript levels of RNAi genes of *A. flavus* in response to AfPV1 infection were measured using qRT-PCR. The expression of most RNAi genes was upregulated by AfPV1 infection, except for RDRP2 (Figure 2), whereas DCLs and AGOs were upregulated the most by AfPV1 infection (Figure 2).

**Figure 3:**
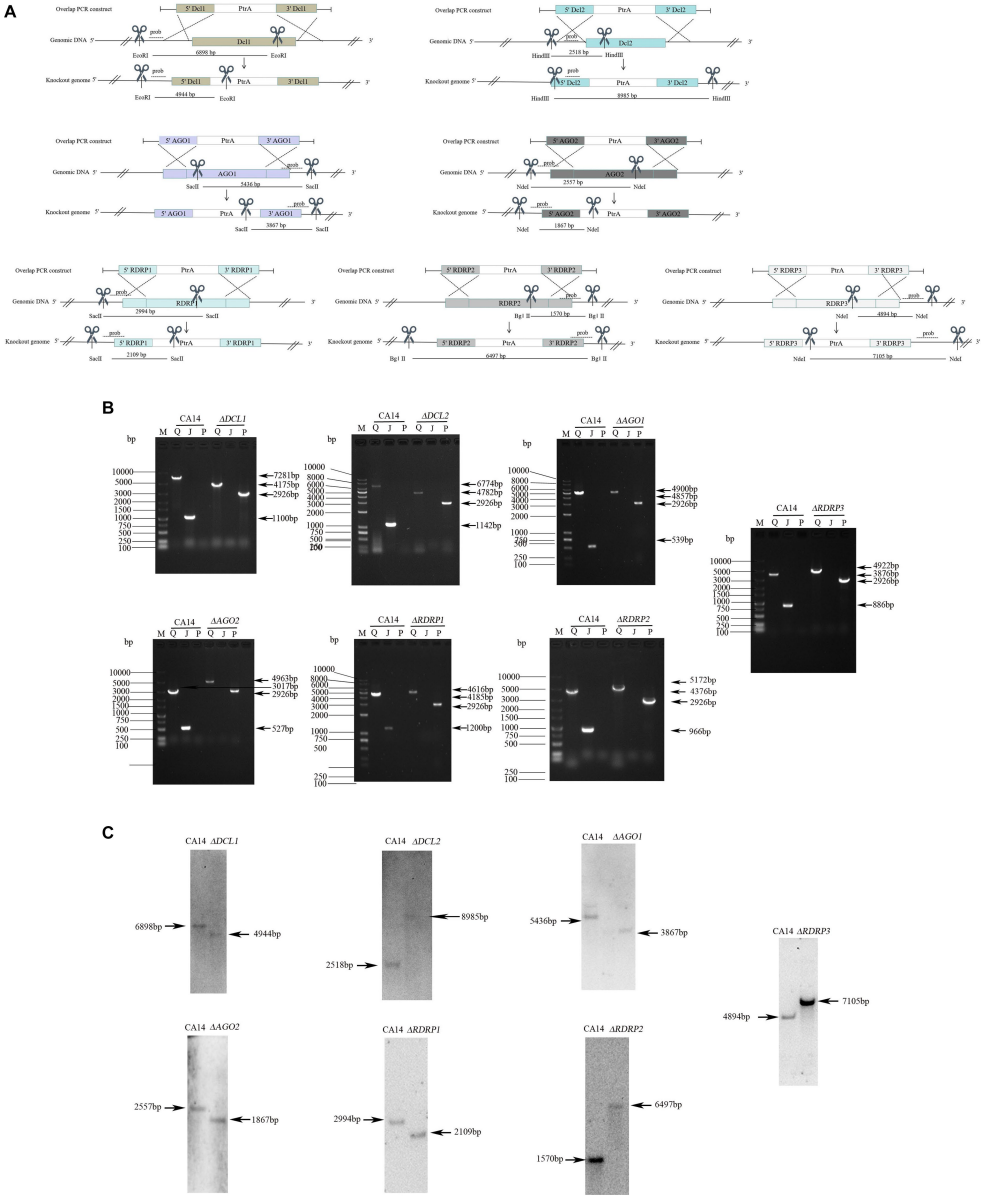
Generation of mutants in *A. flavus*. Schematic diagrams of the homologous recombination strategies used to knock out the RNAi genes **(A)**. Probes and restriction enzymes used for southern blot hybridization are also shown in the schematic diagrams. Each mutant was confirmed by PCR **(B)**. M, DNA molecular marker; Q, the complete sequence segment of the RNAi genes; J, the replaced sequence segment; P, the sequence segment of *ptr*. Southern blot hybridization of the mutants and parent strains **(C)**. The molecular sizes (bp) are indicated on both sides of gels, as well as the size of bands.

To identify the functions of the *A. flavus* RNAi genes in antiviral responses to AfPV1 infection, targeted gene replacement was used to obtain single-gene deletion mutants with deletions of DCL, AGO, and RDRP genes of *A. flavus* strain CA14. And then, the *A. flavus* RNAi gene-deletion mutants were also confirmed by RT-PCR analysis and Southern blotting (Figure 3). The mutants *ΔDCL1*, *ΔDCL2*, *ΔAGO1*, *ΔAGO2*, *ΔRDRP1*, *ΔRDRP2*, and *ΔRDRP3* were successfully obtained, but we failed to generate mutants *ΔDCL3*, and *ΔAGO3*.

**Figure 4:**
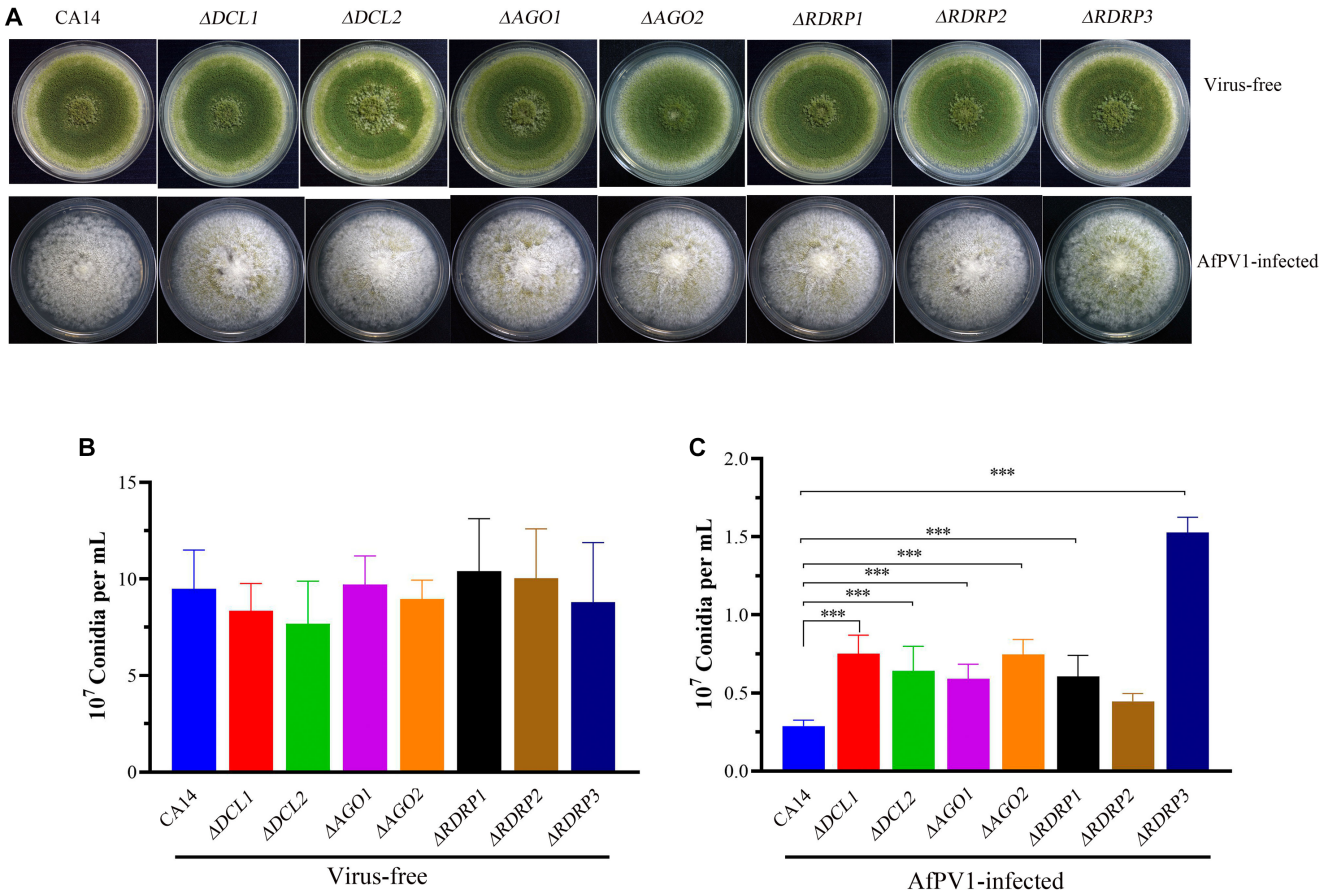
Colony growth of virus-free and AfPV1-infected RNAi mutants and parental strains on PDA for 8 days **(A)**. The sporulation of virus-free **(B)** and AfPV1-infected strain **(C)** on PDA for 8 days. Significant differences by Dunnett’s test are indicated by ∗ (*p* < 0.05), ∗∗ (*p* < 0.01), and ∗∗∗ (*p* < 0.001).

Mycelial morphology of each mutant strain showed no significant differences compared with parental CA14, while mycelial morphology of each AfPV1-infected mutant strain also showed no significant differences compared with AfPV1-infected CA14 (Figure 4A). AfPV1 infection significantly reduced sporulation of *A. flavus*, which has been observed previously (Jiang et al., 2019). Sporulation of each *A. flavus* mutant strain showed no significant differences compared with the parental strain (Figure 4B), but sporulation of each AfPV1-infected RNAi mutant strain was increased compared to the AfPV1-infected parental (Figure 4C).

**Figure 5:**
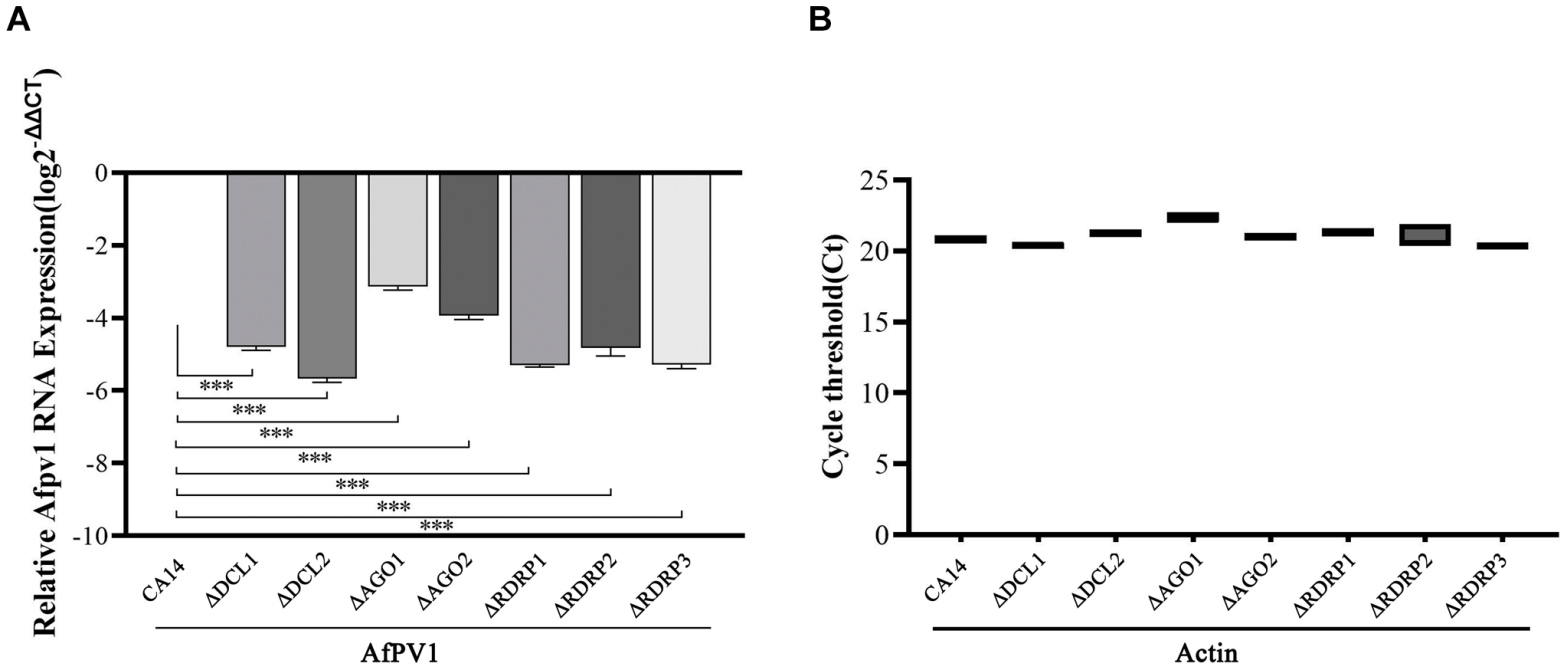
Accumulation of AfPV1 genome in parental and mutant strains **(A)**. The expression of the actin gene is used as a reference in qPCR tests **(B)**. Significant differences in Dunnett’s test are indicated by ∗ (*p* < 0.05), ∗∗ (*p* < 0.01), and ∗∗∗ (*p* < 0.001).

**Figure 6:**
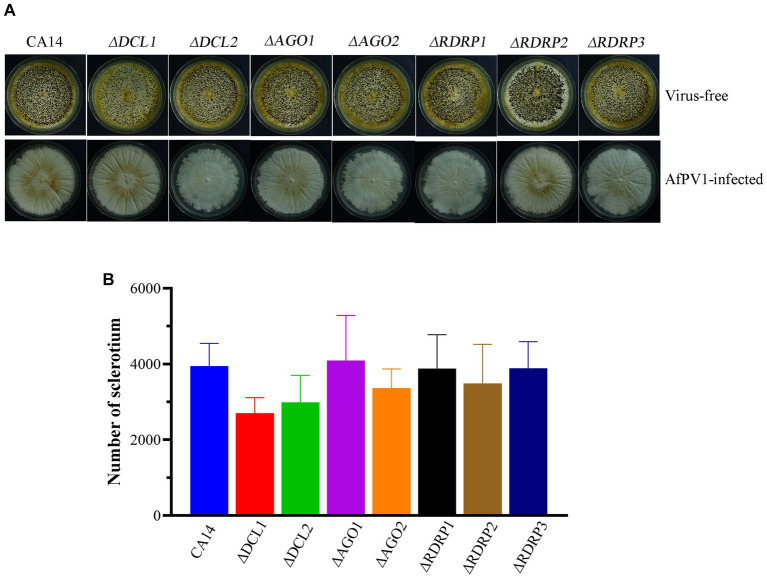
Sclerotia of virus-free and AfPV1-infected RNAi mutants and the parental strain **(A)**. Sclerotial production of virus-free mutants and the parental strain **(B)**.

To investigate RNAi in response to AfPV1 infection, each mutant strain individually infected with AfPV1 was obtained by hyphal anastomosis with an infected strain. And then, the AfPV1 genomic accumulation levels were examined in the AfPV1-infected RNAi mutant strains. AfPV1 infection of RNAi mutant strains significantly decreased AfPV1 accumulation compared with the infected CA14 strain (Figure 5). *A. flavus* produces sclerotia which can survive in unsuitable environments, and CA14 and RNAi mutant could normally produce sclerotia, while AfPV1-infected CA14 and mutant strains produced none (Figure 6).

**Figure 7:**
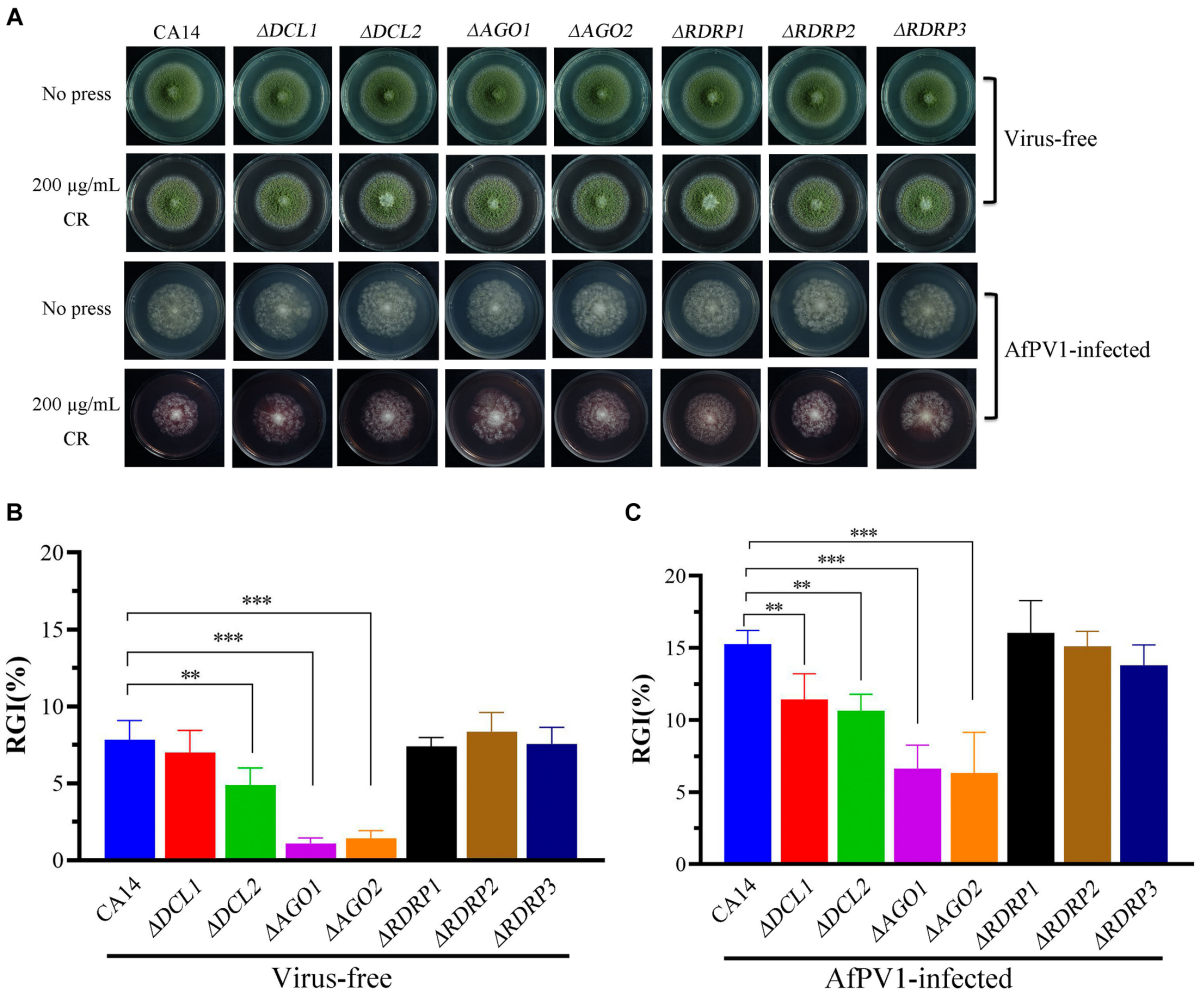
Cell wall stress of virus-free and AfPV1-infected RNAi mutants and the parental strain **(A)**. The RGI of virus-free strains **(B)** and AfPV1-infected strain **(C)** on YGM medium containing 200 μg/μL CR. Significant differences by Dunnett’s test are indicated by ∗ (*p* < 0.05), ∗∗ (*p* < 0.01), and ∗∗∗ (*p* < 0.001).

We also investigated the sensitivity of CA14, mutant strains, and AfPV1-infected CA14 and mutant strains to CR, NaCl, MMS, and H_2_O_2_, which induce cell wall stress, osmotic stress, genotoxic stress, and oxidative stress, respectively. Compared to CA14, *ΔDCL2*, *ΔAGO1*, and *ΔAGO2* mutants showed less sensitivity to cell wall stress, but *ΔDCL1, ΔRDRP1, ΔRDRP2*, and *ΔRDRP3* did not show any differences (Figures 7A,B). Compared with the AfPV1-infected CA14, AfPV1-infected *ΔDCL1, ΔDCL2, ΔAGO1* and *ΔAGO2* mutants showed less sensitivity to cell wall stress (Figures 7A,C).

**Figure 8:**
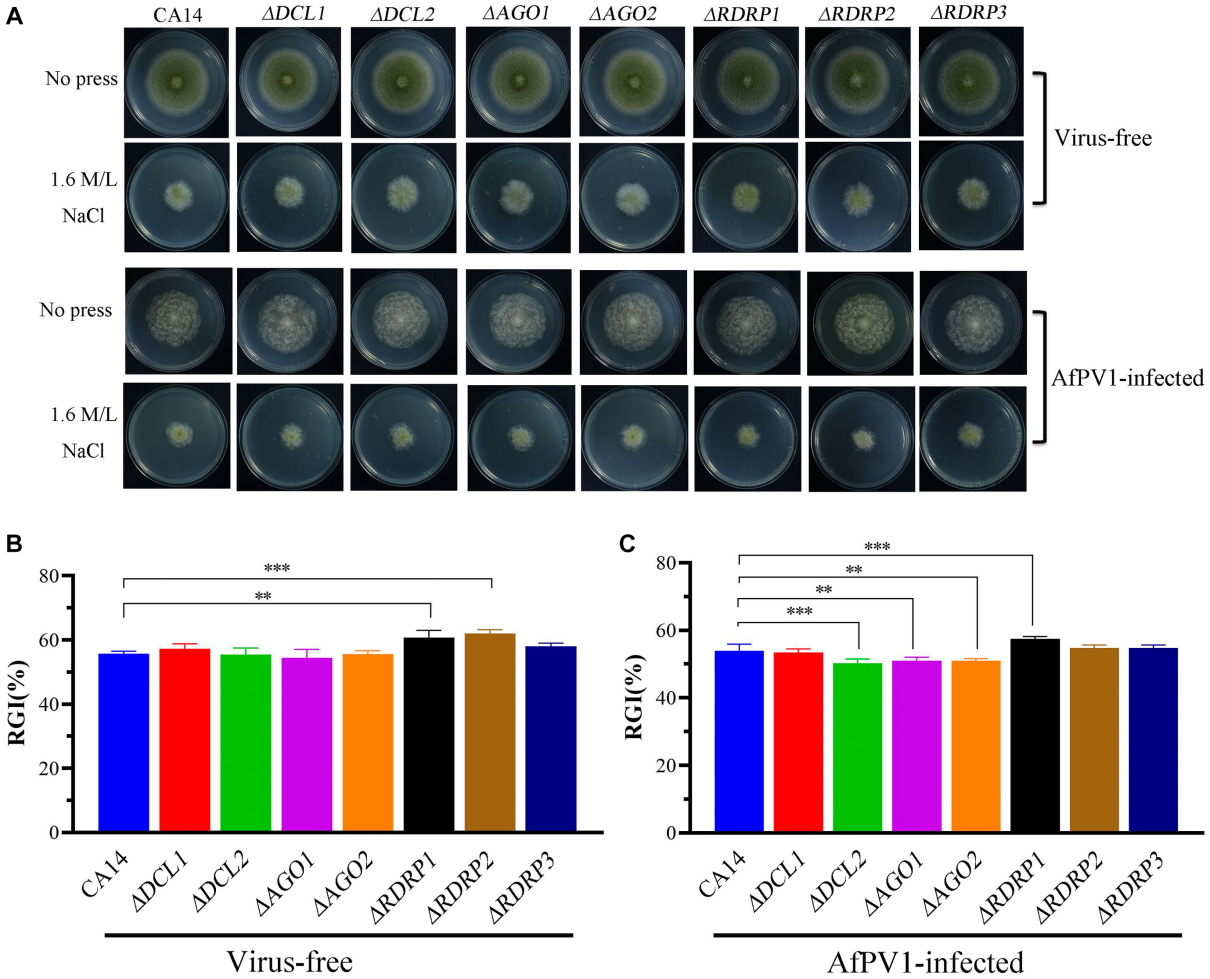
Osmotic stress of virus-free and AfPV1-infected RNAi mutants and the parental CA14 strain **(A)**. The RGI of virus-free strains **(B)** and AfPV1-infected strain **(C)** on YGM medium containing 1.5 M/L NaCl. Significant differences by Dunnett’s test are indicated by ∗ (*p* < 0.05), ∗∗ (*p* < 0.01), and ∗∗∗ (*p* < 0.001).

Compared to CA14, *ΔRDRP1* and *ΔRDRP2* mutants showed more sensitivity, but *ΔDCL1*, *ΔDCL2*, *ΔAGO1*, *ΔAGO2*, and *ΔRDRP3* did not show any differences (Figures 8A,B). Compared with AfPV1-infected CA14, AfPV1-infected mutants (*ΔDCL2*, *ΔAGO1*, and *ΔAGO2*) showed less sensitivity to osmotic stress, but AfPV1-infected mutant *ΔRDRP1* showed more sensitivity (Figures 8A,C).

**Figure 9:**
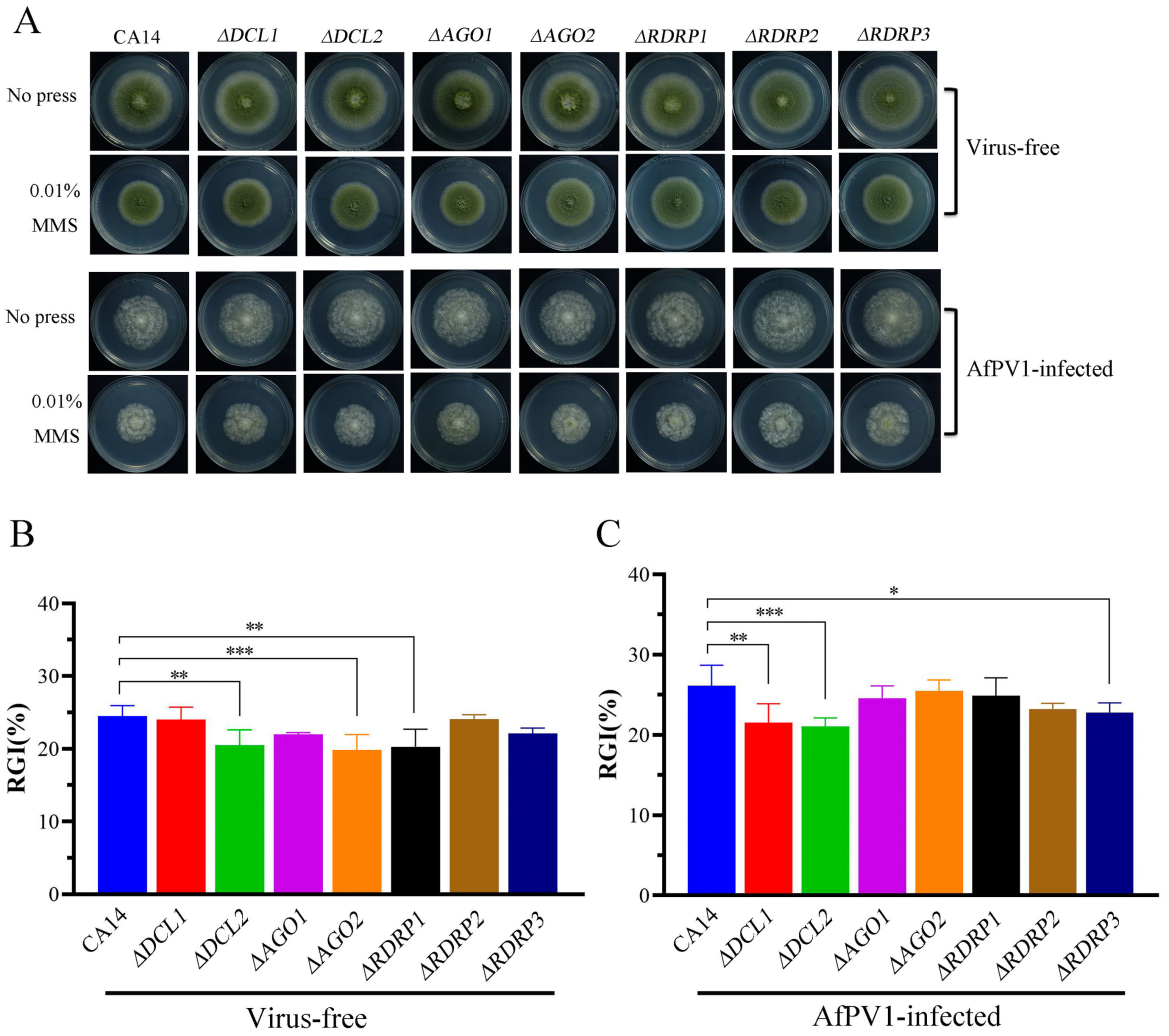
Genotoxic stress of virus-free and AfPV1-infected RNAi mutants and the parental CA14 strain **(A)**. The RGI of virus-free strains **(B)** and AfPV1-infected strain **(C)** on YGM medium containing 0.01% MMS. Significant differences by Dunnett’s test are indicated by ∗ (*p* < 0.05), ∗∗ (*p* < 0.01), and ∗∗∗ (*p* < 0.001).

Compared to CA14, *ΔDCL2*, *ΔAGO2*, and *ΔRDRP1* mutants showed less sensitivity to genotoxic stress, but *ΔDCL1*, *ΔRDRP2*, and *ΔRDRP3* did not show any differences (Figures 9A,B). Compared to AfPV1-infected CA14, the AfPV1-infected *ΔDCL1*, *ΔDCL2*, and *ΔRDRP3* mutants showed less sensitivity to genotoxic stress (Figures 9A,C).

**Figure 10:**
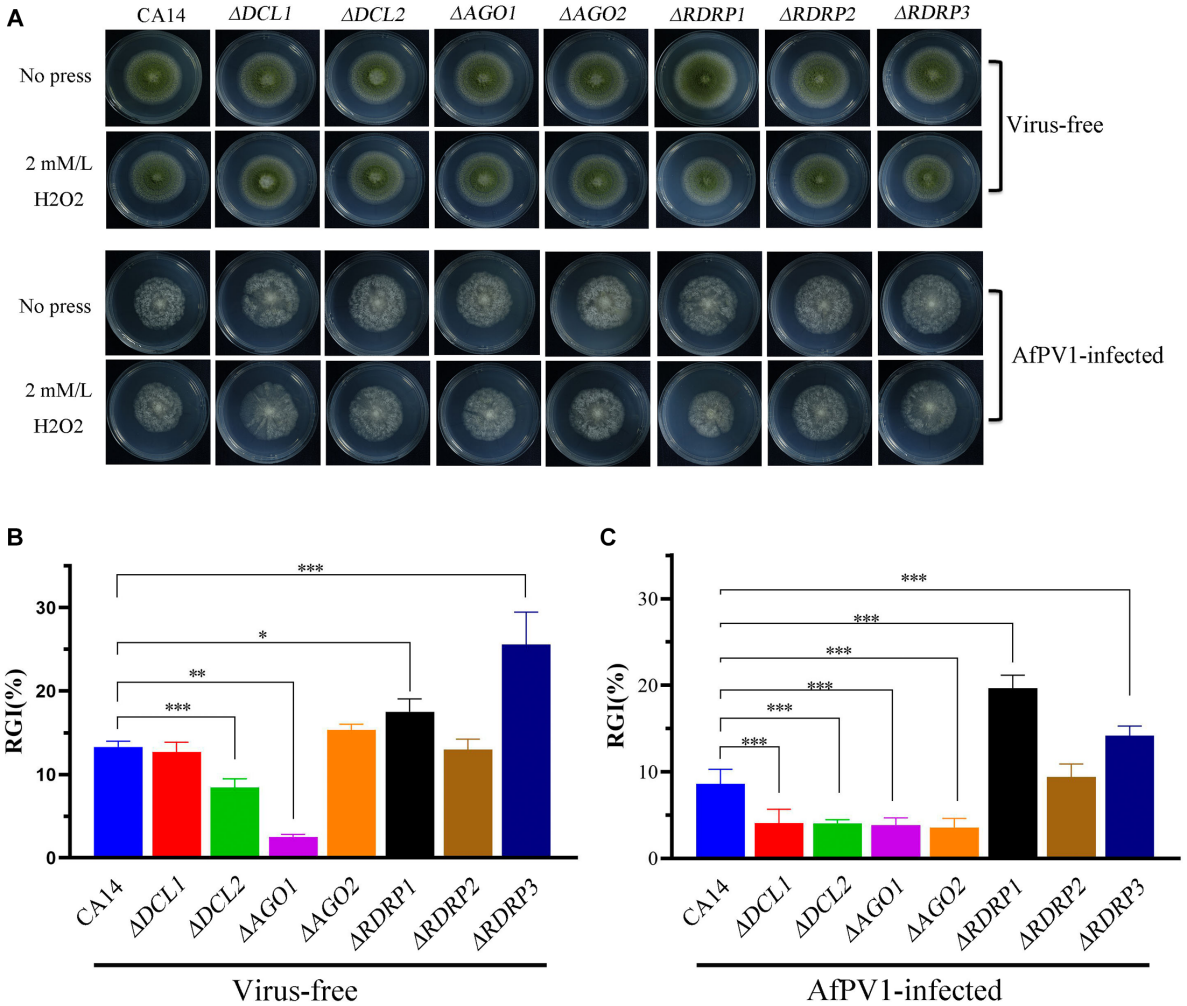
Oxidative stress of virus-free and AfPV1-infected RNAi mutants and the parental CA14 strain **(A)**. The RGI of virus-free strains **(B)** and AfPV1-infected strain **(C)** on YGM medium containing 2 mM/mL H_2_O_2_. Significant differences by Dunnett’s test are indicated by ∗ (*p* < 0.05), ∗∗ (*p* < 0.01), and ∗∗∗ (*p* < 0.001).

Compared to CA14, *ΔDCL2*, and *ΔAGO1* mutants showed less sensitivity to oxidative stress, but *ΔRDRP1* and *ΔRDRP3* mutants showed more sensitivity (Figures 10A,B). Compared to AfPV1-infected CA14, AfPV1-infected *ΔDCL1*, *ΔDCL2*, *ΔAGO1*, and *ΔAGO2* mutants showed less sensitivity to oxidative stress, but AfPV1-infected *ΔRDRP1* and *ΔRDRP2* mutants showed more sensitivity to oxidative stress (Figures 10A,C). These results suggested that the mutants of DCLs and AGOs infected by AfPV1 displayed more changes than RDRP mutants in response to cell wall stress, osmotic stress, and genotoxic stress.

### Analysis of siRNAs derived from AfPV1

**Figure 11:**
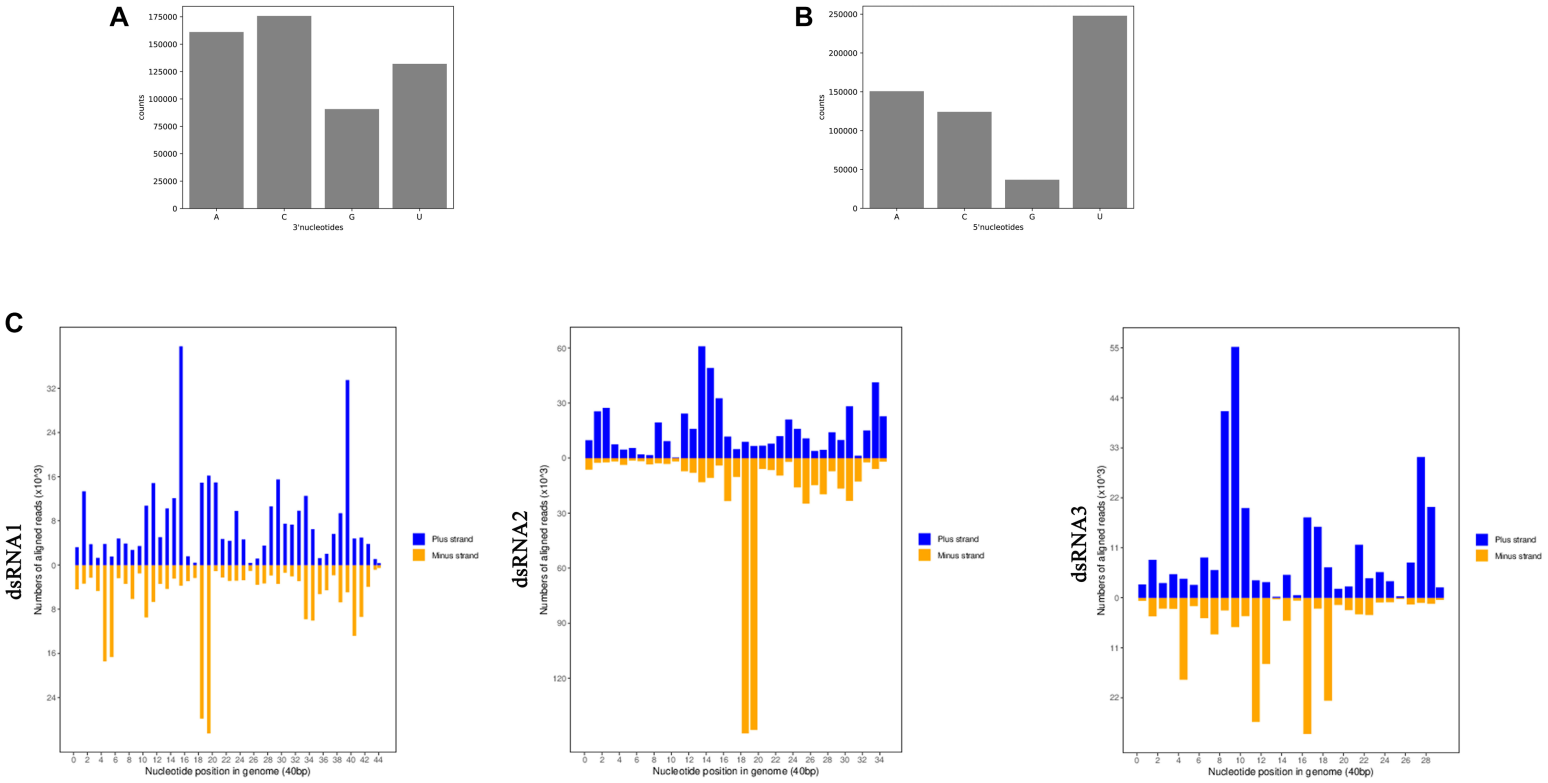
Composition of the 3′-terminal nucleotide **(A)** and 5′-terminal nucleotide **(B)** among vsiRNAs. Profiling of the vsiRNAs on AfPV1 genomes **(C)**.

RNAi has been found associated with the generation of siRNA, and many fungal hosts produce vsiRNA in response to diverse mycoviral infections (Campo et al., 2016; Yu et al., 2018). To examine whether siRNA accumulation is affected by AfPV1 infection, we prepared two isogenic *A. flavus* strains: LD-F1 (virus-free strain), and LD-F1-b (infected with AfPV1). Deep sequencing raw reads from three replicates each averaged 54.25 M for LD-F1 and 50.24 M for LD-F1-b libraries. After sequencing, the data was subjected to adaptor removal, and the low-quality tags and contaminants due to adaptor ligation were also filtered out. Clean reads, based on three replicates each, consisted of 48.84 M and 43.48 M from LD-F1 and LD-F1-b libraries, respectively. The number of unique clean reads was 16.73 M for LD-F1 and 4.26 M for LD-F1-b (Table 1). Therefore, AfPV1 infection reduced the unique reads of sRNAs in *A. flavus*. miRNA, rRNA, anoRNA, snRNA, tRNA, and unknown RNA were found in uninfected LD-F1. And in LD-F1-b, vsiRNA was 18.46% among all sRNA reads. To observe the genomic distribution of vsiRNA, the 5′-terminus of sRNA from AfPV1 was mapped to the corresponding genome of AfPV1 (dsRNA1 accession MK344768, dsRNA2 accession MK344769 and dsRNA3 accession MK344770 in GenBank) based on their polarities and genomic locations (Figure 11C). The vsiRNA derived from AfPV1 was 15–41 nt in length, and most vsiRNA was 20 nt. The 5′-terminal nucleotide composition of AfPV1-derived vsiRNA revealed an obvious preference for uridine (U) residues, while guanidine (G) was the least abundant (Figure 11B), but there was no preference in the 3′-terminal nucleotides (Figure 11A).

**Table 1:** Read statistics from sRNA sequencing of virus-free (LD-F1) and AfPV1-infected (LD-F1-b) strains of *A. flavus*.

Sample	RawReads	CleanReads	CleanReads_uniq
LD-F1_1	17.82M	16.42M	5.83M
LD-F1_2	17.85M	15.35M	5.06M
LD-F1_3	18.58M	17.07M	5.84M
LD-F1-b_1	16.59M	14.75M	1.43M
LD-F1-b_2	16.03M	13.99M	1.37M
LD-F1-b_3	17.62M	14.74M	1.46M

LD-F1_1, LD-F1_2, and LD-F1_3 are three replicates of LD-F1 strain; LD-F1-b_1, LD-F1-b_2, and LD-F1-b_3 are three replicates of LD-F1-b strain.

### Small RNAs are differentially expressed in AfPV1-infected and virus-free *Aspergillus flavus*

**Figure 12:**
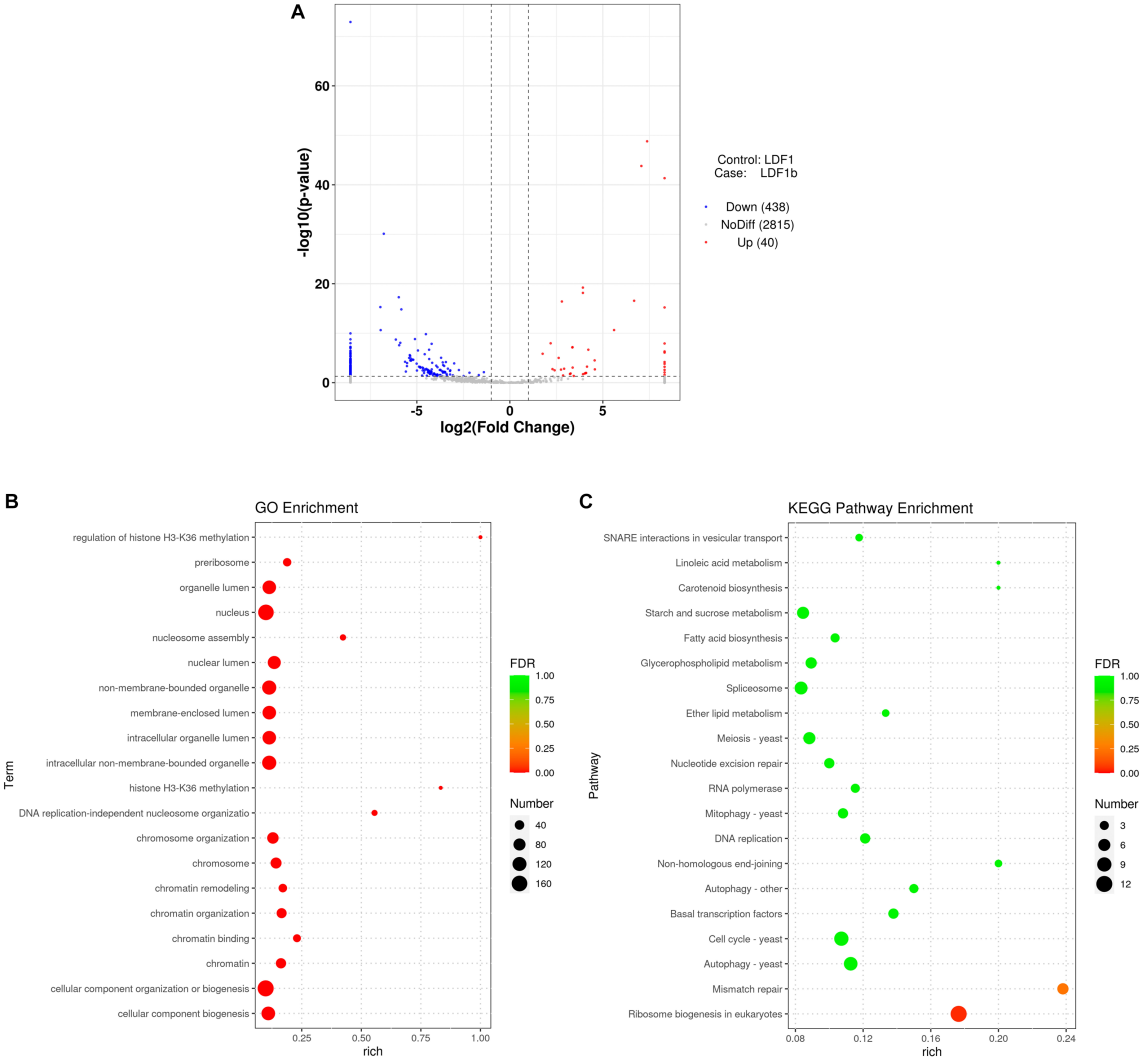
Different miRNA expression between AfPV1-infected strain (LD-F1-b) and virus-free strain (LD-F1) **(A)**. GO term **(B)** and KEGG pathway **(C)** enrichment of the different miRNAs.

Because AfPV1 infection resulted in abnormal colony morphology and hypovirulence of *A. flavus*, we investigated the functions of sRNA affected by AfPV1 infection. Compared with virus-free LD-F1, the AfPV1-infected LD-F1-b showed upregulation of 40 miRNAs and downregulation of 438 miRNAs (Figure 12). Moreover, when some miRNAs were randomly selected, and the expression was analyzed by stem-loop RT-PCR, the results showed agreement with sequencing analysis (Supplementary Figure S2). Non-membrane-bounded organelle (GO:0043228), intracellular non-membrane-bounded organelle (GO:0043232), and membrane-enclosed lumen (GO:0031974) were found after GO enrichment (Figure 12). SNARE interactions in vesicular transport, mitophagy, and autophagy were found in KEGG pathway enrichment (Figure 12). These terms and pathways were closely related to vacuole production in eukaryotic cells^9^.

## Discussion

RNAi is known to be one of the most important antiviral response mechanisms in organisms (Baulcombe, 2022). Moreover, the types of RNAi components are similar in fungi, plants, and animals (Sioud, 2021). To start the process, virus infection stimulates host RNAi reaction, long dsRNA is cut into siRNA for RISC assembling, which is responsible for target RNA cleavage, and therefore siRNA is a critical component for RNAi in organisms (Mengistu and Tenkegna, 2021; Baulcombe, 2022). We found three DCL proteins, three AGO proteins, and three RDRP proteins in *A. flavus*, and AfPV1 infection upregulated DCL1, DCL2, DCL3, AGO1, AGO2, AGO3, RDRP1, and RDRP3, but did not affect RDRP2 (Figure 2). The expression levels of DCL2, AGO1, RDRP3, RDRP4, and RDRP5 were significantly increased by FgV2 and FgV3 infection (Yu et al., 2018). *Fusarium graminearum* Hypovirus 2 (FgHV2) infection significantly upregulated the expression levels of DCL2, AGO1, and RDRP3 in *F. graminearum* (Li et al., 2015). In *C. parasitica*, DCL2 and AGO2 were upregulated by CHV1 and mycoreovirus 1 (MyRV1) infections (Segers et al., 2007; Xuemin et al., 2008). Our results suggested that the dsRNA antiviral responses found in *A. flavus* were similar to those in *F. graminearum* and *C. parasitica*. Most of the single component RNAi mutants of *F. graminearum* did not alter viral RNA accumulation (Yu et al., 2018), but we observed that AfPV1 accumulation was significantly decreased in AfPV1-infected single-component RNAi mutants compared with AfPV1-infected CA14 (Figure 4). This result does not follow the paradigm that RNAi acts as an antiviral mechanism. In *F. graminearum*, double-knockout mutants (*ΔDCL1/DCL2* and *ΔAGO1/ΔAGO1*), showed significantly increased accumulation of viruses FgV1 and FgV2 (Yu et al., 2018). Future studies are needed with multiple knockout mutants of RNAi genes in *A. flavus* to see their effects on viral accumulation. In previous studies, AfPV1 caused phenotypic changes and hypovirulence in *A. flavus*, and found that it had the potential for treatment of *A. flavus* infections (Jiang et al., 2019, 2022). To study antiviral responses, single-gene deletion mutants of *A. flavus* were constructed. In our study, the mutants of DCLs and AGOs infected by AfPV1 displayed more changes than RDRP mutants in response to cell wall stress, osmotic stress, and genotoxic stress (Figures 7–9). Moreover, transcripts of DCLs and AGOs were also altered more than those of RDRP genes following AfPV1 infection (Figure 2). DCLs and AGOs have been shown to be involved in the redundant functional role of antiviral RNAi in *N. crassa*, *C. parasitica* and *F. graminearum*, while the RDRPs are dispensable for antiviral RNAi (Zhang et al., 2014; Yu et al., 2018, 2020; Honda et al., 2020). However, we did not obtain the single-gene deletion mutants *ΔDCL1* or *ΔDCL3*, and further study of such mutants infected by AfPV1 are required.

sRNA sequencing can be used for research on fungal defense against viruses as well as counter-defense research (Donaire and Ayllón, 2017; Özkan et al., 2017). The virus-derived sRNA in fungi may stimulate similar RNAi pathways to those found in plants and animals (Wang et al., 2022a). In our study, AfPV1 infection reduced the number of unique reads of sRNAs in *A. flavus* (Table 1). These results suggested that AfPV1 may suppress RNA silencing in *A. flavus*, and therefore AfPV1 may encode an RNA silencing suppressor (RSS). Currently, there are several identified RSSs found in mycoviruses, namely p29 encoded by CHV1, p20 encoded by Fusarium graminearum hypovirus 1 (FgHV1), s10 encoded by RnMyRV3, ORF2 encoded by FgV1, and p24 encoded by CHV4 (Qihong et al., 2009; Yaegashi et al., 2013; Yu et al., 2020; Aulia et al., 2021; Wang et al., 2022b). And recently, the capsid protein of a partitivirus has been identified as an RSS in plants and fungi (Shimura et al., 2022). The AfPV1 genome has three segments (dsRNA1 accession MK344768, dsRNA2 accession MK344769, and dsRNA3 accession MK344770), a dsRNA1-encoded viral RDRP protein, and a dsRNA2-encoded viral capsid protein (CP), but the dsRNA3 encodes a protein without known similarity in the NCBI database (Jiang et al., 2019). Therefore, we speculate that the dsRNA2 or dsRNA3 encodes a protein that may function as an RNA silencing suppressor, but this needs further study. In plants and animals, there is a preference for the 5′-terminal nucleotides of sRNA, when loading AGOs (Eggleston, 2009). For example, *Arabidopsis* AGO2 and AGO4 mainly bind sRNA beginning with adenosine (A) at the 5′-terminal, whereas AGO1 preferentially binds miRNA such as uridine (U) at the 5′-terminal (Deleris et al., 2006). In this study, U residues were the most common at the 5′-terminal position of AfPV1-derived vsiRNAs (Figure 11B). The functional AGOs of *A. flavus* needed further exploration. In a previous study, AfPV1 infection resulted in several large vacuoles in *A. flavus* cells (Jiang et al., 2022). Here, we examined functions of sRNA affected by AfPV1 infection, and found that some GO terms and KEGG pathways are closely related to vacuole production in eukaryotic cells (Figure 12), and support the previous finding that AfPV1 effects are related to vacuole formation in *A. flavus* cells.

In conclusion, we found that RNAi components of *A. flavus* participated in the antiviral response against AfPV1 infection. AfPV1 infection upregulated the expression of the RNAi components in *A. flavus* but reduced the number of unique reads of sRNAs in *A. flavus*. The knockout mutants for each RNAi component caused a decrease of AfPV1 RNA accumulation. The mutants of DCLs and AGOs infected by AfPV1 displayed more changes than RDRP mutants in response to cell wall stress, osmotic stress, and genotoxic stress. We also found that some GO terms and KEGG pathways for functions of virus-induced sRNA were closely related to vacuole production in eukaryotic cells, which is consistent with previous findings that the major effects of AfPV1 infection in *A. flavus* cells are related to vacuole formation.

## Data availability statement

The datasets presented in this study can be found in online repositories. The names of the repository/repositories and accession number(s) can be found in the article/Supplementary material.

## Author contributions

YinJ and XQ: conceptualization. YinJ, XL, XT, and JZ: data curation. XL, QW, BW, WY, YanJ, and XQ: formal analysis. YinJ, XL, and TH: methodology. YinJ: roles and writing – original draft. TH: writing – review & editing. All authors contributed to the article and approved the submitted version.

## Footnotes

^1^ https://www.ncbi.nlm.nih.gov/guide/domains-structures/

^2^ https://rfam.xfam.org/

^3^ http://www.mirbase.org/

^4^ https://sourceforge.net/projects/mireap/

^5^ http://www.targetscan.org/cgibin/targetscan/data_download.cgi?db=vert_61

^6^ https://www.ncbi.nlm.nih.gov/

^7^ http://www.geneontology.org/

^8^ http://www.genome.jp/kegg

^9^ https://www.kegg.jp/kegg-bin/show_pathway?map04130/K08502%09%23FFFFFF,red/K08515%09%23FFFFFF,red/
