# Corrigendum: RNA interference of *Aspergillus flavus* in response to Aspergillus flavus partitivirus 1 infection

**DOI:** 10.3389/fmicb.2024.1546815

**Published:** 2025-01-07

**Authors:** Yinhui Jiang, Xiang Liu, Xun Tian, Jianhong Zhou, Qinrong Wang, Bi Wang, Wenfeng Yu, Yanping Jiang, Tom Hsiang, Xiaolan Qi

**Affiliations:** ^1^Key Laboratory of Endemic and Ethnic Diseases, Ministry of Education, Guizhou Medical University, Guiyang, China; ^2^Key Laboratory of Medical Molecular Biology of Guizhou Province, Guizhou Medical University, Guiyang, China; ^3^Department of Dermatology, The Affiliated Hospital, Guizhou Medical University, Guiyang, China; ^4^School of Environmental Sciences, University of Guelph, Guelph, ON, Canada

**Keywords:** *Aspergillus flavus*, mycoviruses, RNA-dependent RNA polymerase, dicer, argonaute, antiviral response, small RNA

In the published article, there was an error in Figure 9 as published. The methyl methanesulfonate (0.01% MMS) treated AfPV1-infected Δ*RDRP2* and Δ*RDRP3* mutants were for the same picture (since they are almost identical) in Figure 9. The corrected Figure 9 and its caption appear below.

**Figure 9:**
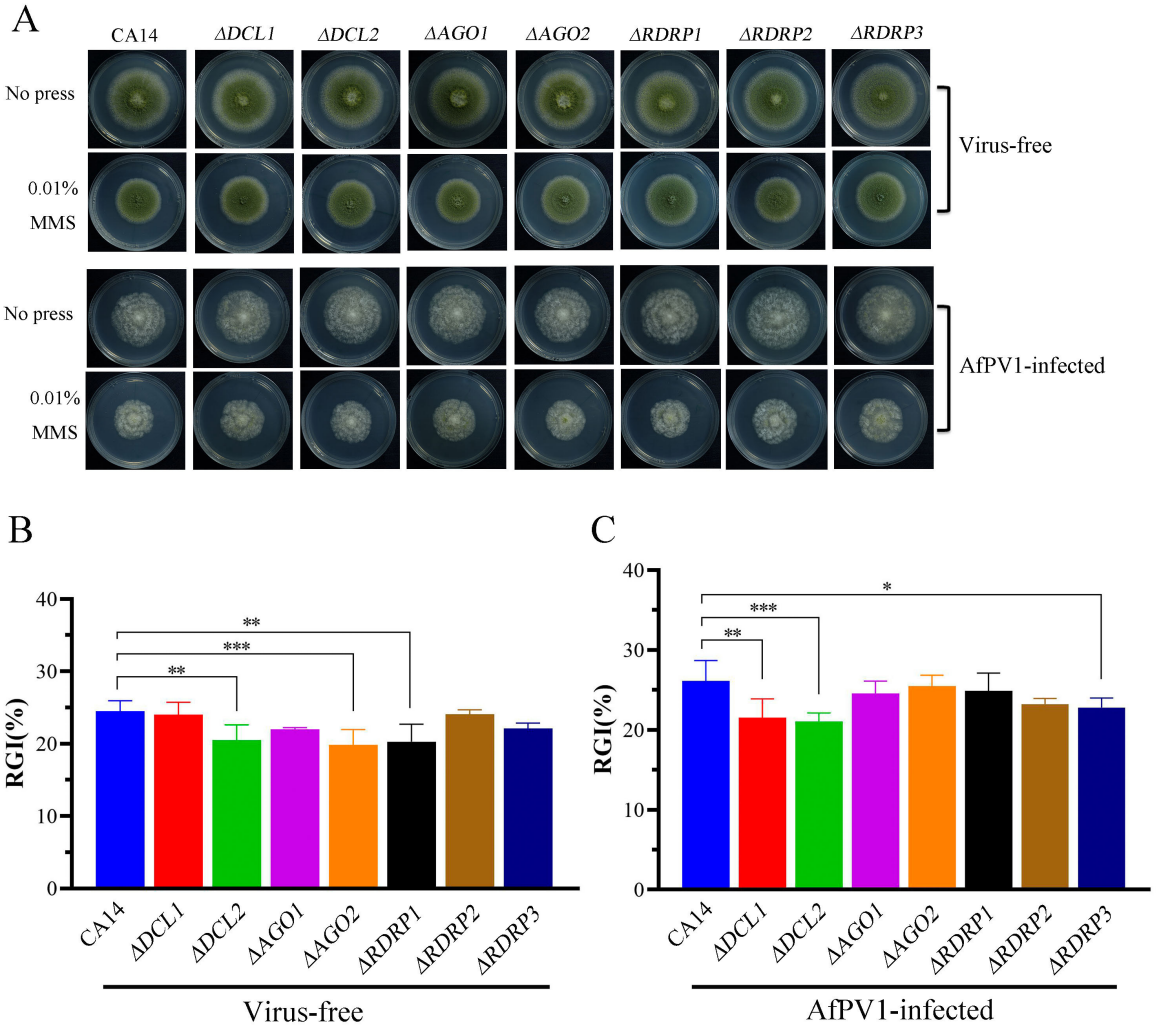
Genotoxic stress of virus-free and AfPV1-infected RNAi mutants and the parental CA14 strain **(A)**. The RGI of virus-free strains **(B)** and AfPV1-infected strain **(C)** on YGM medium containing 0.01% MMS. Significant differences by Dunnett's test are indicated by * (*p* < 0.05), ** (*p* < 0.01), and *** (*p* < 0.001).

The authors apologize for this error and state that this does not change the scientific conclusions of the article in any way. The original article has been updated.

