# Supplementary material for: RNA interference of Aspergillus flavus in response to Aspergillus flavus partitivirus 1 infection
Source: Front Microbiol. 2023 Nov 14;14:1252294. doi: 10.3389/fmicb.2023.1252294 (PMC10682719; doi:10.3389/fmicb.2023.1252294)
Supplement: Supplementary file 1 [file Table_1.docx]

Table S1 The primers used in this study

| Name | Secquence（5'- 3'） |
| --- | --- |
| DCL1-qf | TTCGGGAGGCCGAAAAACTT |
| DCL1-qr | GCGAGAATAGCAAGGGCAGA |
| DCL2-qf | TCAAATGGGACTGGCTTGCT |
| DCL2-qr | GTCCCGGTATCCATAGCAACA |
| AGO1-qf | TTTCAGAGGTCGTGGTCGTG |
| AGO1-qr | GCCCTCCTGGACTCGTGTAT |
| AGO2-qf | CCAGATTGGACACAAGGCAC |
| AGO2-qr | AAACTGCGGGGAACAATGAA |
| RDRP1-qf | GACTTTGTCGGGGAGCAGTT |
| RDRP1-qr | GGCACATCAGAGAGGGTAAAGTC |
| RDRP2-qf | AATCCGCTTCAAACCACCTC |
| RDRP2-qr | GACCGGACTAGCGATCTGAG |
| RDRP3-qf | CTCGAACTTTCCGCCCTCTT |
| RDRP3-qr | CCTTCAGGATCACGCCAGTT |
| AfPV-det1 | GTTACACCACGCCAAAAGCAA |
| AfPV-det2 | GAAATCCAGACCTACACCAAA |
| dsRNA2-F | AACCCTTCACCAACTTCACAAA |
| dsRNA2-R | GCATCAAGACAACCTTCAAACA |
| Afactin-F | ACGGTGTCGTCACAAACTGG |
| Afactin-R | CGGTTGGACTTAGGGTTGATAG |
| DCL1-1F | CAGTTGCGATATCTTCCAGC |
| DCL1-1R | CGAGGTGCCGTAAAGCACTAAGGAAAGGTAAAATGACTGG |
| DCL1-2F | CTGGATGGAGGCGGATAAAGTATTGGAAGG GTGGTTGGTG |
| DCL1-2R | TGCGAAGGTATTGGGAGTG |
| DCL2-1F | AGCAGGTGAAGGAGTTTGATA |
| DCL2-1R | GTGCCGTAAAGCACTAAGAGGGACTGGTGTTTGG |
| DCL2-2F | ATGGAGGCGGATAAAGTTCCAAGGGACGACTGTAT |
| DCL2-2R | CGAAGGTGTTATGGTAGCG |
| AGO1-1F | CCACACCGAACTATATGTAG |
| AGO1-1R | GAGGTGCCGTAAAGCACTAAAGAGGTGAAACCATAAGTGT |
| AGO1-2F | TGGATGGAGGCGGATAAAGTCTCCACTTGACAGCTGTACC |
| AGO1-2R | GTGAGAACGGTCGAGAAGA |
| AGO2-1F | ATGGAGGAATTCGAAGTCGT |
| AGO2-1R | GAGGTGCCGTAAAGCACTAACGATGATGTACTTGCTCGCA |
| AGO2-2F | TGGATGGAGGCGGATAAAGTGATACCTTTGGTGGACGCTA |
| AGO2-2R | CTAGATGTACCAAACTGCGA |
| AGO3-1F1 | ATGTCGGTAACATAGTCGAC |
| AGO3-1R | GAGGTGCCGTAAAGCACTAAAGGGAAAGAAGATGACGTAT |
| AGO3-2F | CTGGATGGAGGCGGATAAAGTTGGCGAAGAAAGGTCAAAC |
| AGO3-2R | TTCTCGTGAACAAGCACATC |
| RDRP1-1F1 | CGGCACGTTTTGAGATCGT |
| RDRP1-1R | GAGGTGCCGTAAAGCACTAAGATTGGACTTGAGCAGCATG |
| RDRP1-2F | TGGATGGAGGCGGATAAAGTTGCGAATCCGACAGTCAGG |
| RDRP1-2R2 | CATATCAATGGCGCTGGTTG |
| RDRP2-1F | ATGTCACATAATCGTCGTGG |
| RDRP2-1R | CGAGGTGCCGTAAAGCACTAAGGGTAAACCGATCTGTGTC |
| RDRP2-2F | CTGGATGGAGGCGGATAAAGTGATTGAGGTCCCGGATGAC |
| RDRP2-2R | TTAGAACCCTAGCAGTTCGT |
| RDRP3-1F | ATGGCGTTTCCTCACACC |
| RDRP3-1R | GAGGTGCCGTAAAGCACTAAATGGCCCGTAGTGAAGGAG |
| RDRP3-2F | GGATGGAGGCGGATAAAGTCACTTTGGTCTTGAGGAACA |
| RDRP3-2R | TCACTCCACGGAGAGATCA |
| Ptr-F | TTAGTGCTTTACGGCACCTCG |
| Ptr-R | ACTTTATCCGCCTCCATCCAG |
| det-DCL1-F | GGCTGATGGTGATGCTCTTA |
| det-DCL1-R | ATGGTCTTGTCTTACGGTCG |
| det-DCL2-F | GAAAGTCCGTCGCATCCAA |
| det-DCL2-R1 | TCTGGACAATAGTGGTGCG |
| det-AGO1-F1 | CAAGACCAGCACCGAGGTCC |
| det-AGO1-R1 | ATGGTCTTGTCTTACGGTCG |
| det-AGO2-F | TACCAGACCTCGCAGATGAT |
| det-AGO2-R | GGTATGTGAACGAGTTCTTGT |
| det-AGO3-F | TCAACTCCGAATACCCTCCT |
| det-AGO3-R | CCTGATTGCTGGCGTCTCT |
| det-RDRP1-F | CCTTGAGAAACCTTGCTGTC |
| det-RDRP1-R | CCTTCATCCCACCACTAGC |
| det-RDRP2-F1 | GACGAGAAGCTGCTAGGTCG |
| det-RDRP2-R1 | CCTTGGTCGACAGCAATCCT |
| det-RDRP3-F | TGCTGCGAGAAAGTATAGCC |
| det-RDRP3-R | GAAGCATTGCAGCGCTACTG |
| DCL1-prob-F | CTATGGACGGAGGAGTTAG |
| DCL1-prob-R | GAGATGCGATCATCGAGAC |
| DCL2-prob-F | AGCTTCAACTATCCCTATAAAT |
| DCL2-prob-R | GGTTGGAGTAGAGAGAGGT |
| AGO1-prob-F | CTAGTGCGATTGCCATTGAG |
| AGO1-prob-R | GTACACGAGGCAGCATCTC |
| AGO2-prob-F | CTGTCCATCCATGTGGGAAG |
| AGO2-prob-R | GGCTGAGTCAAACAAGATAC |
| RDRP1-prob-F | GTGACGATATAGCTCAAGTC |
| RDRP1-prob-R | CGCAAGGAGGTATACGGTGA |
| RDRP2-prob-F | GGACTAGCGATCTGAGGTTC |
| RDRP2-prob-R | CATACCAGACTGGTGTCAGTA |
| RDRP3-prob-F | GCATCAAGATCATGCTACTCC |
| RDRP3-prob-R | CGCAGGACCATCTCATGATAT |
| aof-m12156-RT | GTCGTATCCAGTGCAGGGTCCGAGGTATTCGCACTGGATACGACTCCATC |
| aof-m12156-F | CCAGGCCACTCTGTAGATGTCG |
| ata-m5084-RT | GTCGTATCCAGTGCAGGGTCCGAGGTATTCGCACTGGATACGACGATCCT |
| ata-m5084-F | ACGAGATCACCATACGGTACTGC |
| aly-m163-RT | GTCGTATCCAGTGCAGGGTCCGAGGTATTCGCACTGGATACGACGATCGA |
| aly-m163-F | AACACGCTGAAGAGGACTTGGAA |
| aly-m3441-RT | GTCGTATCCAGTGCAGGGTCCGAGGTATTCGCACTGGATACGACTTCCTT |
| aly-m3441-F | GCGCGTTCAAAGCATCTTTG |
| miRNA-R | ATCCAGTGCAGGGTCCGAGG |
| 5SrRNA-F | TAGGGTGTGGAGAACAGGGCTTC |
| 5SrRNA-R | AGGAGGGATTCGCTGGTGGTC |
