# Supplementary material for: RNA interference of Aspergillus flavus in response to Aspergillus flavus partitivirus 1 infection
Source: Front Microbiol. 2023 Nov 14;14:1252294. doi: 10.3389/fmicb.2023.1252294 (PMC10682719; doi:10.3389/fmicb.2023.1252294)
Supplement: Supplementary file 2 [file Table_2.docx]

Table S2. RNAi component proteins selected in *Aspergillus*

| DCL1 | DCL2 | AGO1 | AGO2 | RDRP1 | RDRP2 | RDRP3 |
| --- | --- | --- | --- | --- | --- | --- |
| *A. flavus* (QRD84710.1) | *A. flavus* (UDD63567.1) | *A. flavus* (RAQ49550.1) | *A. flavus* (XP_041143074.1) | *A. flavus* (KAF7629688.1) | *A. flavus* (QRD94454.1) | *A. flavus* (XP_041148631.1) |
| *A. oryzae* (KDE83102.1) | *A. oryzae* (XP_001818742.1) | *A. oryzae* (XP_023089033.1) | *A. oryzae* (XP_001819808.1) | *A. oryzae* (XP_001826725.3) | *A. oryzae* (EIT72389.1) | *A. oryzae* (OOO08449.1) |
| *A. transmontanensis* (KAE8314127.1) | *A. minisclerotigenes* (KAB8269355.1) | *A. minisclerotigenes* (KAB8273159.1) | *A. parasiticus* (KAB8200969.1) | *A. parasiticus* (KAB8207520.1) | *A. arachidicola* (PIG89920.1) | *A. transmontanensis* (KAE8313797.1) |
| *A. parasiticus* (KAB8202836.1) | *A. parasiticus* (KAB8199093.1) | *A. parasiticus* (KAB8212225.1) | *A. sergii* (KAE8328805.1) | *A. pseudonomiae* (XP_031946040.1) | *A. transmontanensis* (KAE8306601.1) | *A. parasiticus* (KAB8202988.1) |
| *A. novoparasiticus* (KAB8225262.1) | *A. arachidicola* (KAE8343078.1) | *A. sergii* (KAE8322814.1) | *A. tamarii* (KAE8161111.1) | *A. pseudocaelatus* (KAE8420364.1) | *A. parasiticus* (KAB8203019.1) | *A. caelatus* (XP_031922919.1) |
| *A. lentulus* (GFF65161.1) | *A. tamarii* (KAE8159818.1) | *A. transmontanensis* (KAE8309012.1) | *A. caelatus* (XP_031924479.1) | *A. caelatus* (XP_031932025.1) | *A. caelatus* (XP_031922440.1) | *A. tamarii* (KAE8167765.1) |
| *A. fumigatus* (KAH3477776.1) | *A. caelatus* (XP_031920672.1) | *A. novoparasiticus* (KAB8219177.1) | *A. fumigatus* (KAH1496145.1) | *A. terreus* (GES64775.1) | *A. fumigatus* (KAH1293138.1) | *A. lentulus* (GAQ09439.1) |
|  | *A. fumigatus* (KAH1340517.1) | *A. caelatus* (XP_031928919.1) | *A. lentulus* (KAF4158731.1) | *A. lentulus* (KAF4169051.1) |  | *A. fumigatus* (KAH1491884.1) |
|  |  | *A. tamarii* (KAE8166881.1) | *A. niger* (XP_001399795.2) | *A. niger* (KAI2938209.1) |  |  |
|  |  | *A. lentulus* (GFG10102.1) |  |  |  |  |
|  |  | *A. fumigatus* (KAH1292139.1) |  |  |  |  |
