# Supplementary material for: RNA interference of Aspergillus flavus in response to Aspergillus flavus partitivirus 1 infection
Source: Front Microbiol. 2023 Nov 14;14:1252294. doi: 10.3389/fmicb.2023.1252294 (PMC10682719; doi:10.3389/fmicb.2023.1252294)
Supplement: Supplementary file 3 [file Table_3.docx]

>probe_DCL1

CTATGGACGGAGGAGTTAGAGGAACACTTGATGGCCGGCAATGTGGACTTCATTGTGCACTCTTTGAAAGGTTTGCTATCTGCGTATGGTAGCGGTAATACTGCAGTGGACGCTGATAAAGTGACTGTCCTCAGATGTACCCACTACCCTACCCCCCGCGTGTACGCTGGGCCCTATGATGGAGCGTGAAGATTCCAGGGATGTACTGGTCATCAAGCAAGGGCTACCGAACATGTCTTTATCCGATTTGCCTGCAGGATCTGTCGTTGGCACCTCCTCCATCCGCCGTACTGCTCAATTGGCCTTGAAATACCCTCACCTAAAGGTTATTGATGTGCGTGGGAATATCGGCACACGACTCGCTAAACTAGACGCGGAAGATTCACCTTATACTTGCATTATCCTGGCAGCTGCCGGCTTACTGAGACTAGGTCTCGATGATCGCATCTC

>probe_DCL2

AGCTTCAACTATCCCTATAAATTGTCAAGGAACGCCCCTAACCAAACAAAGAACAAAAATAAACCAAAGACCCAAACCCAGTGAAAATAAAGGAGACAAAAGCCCAAAAAGCCCAAACAGAACCCCTGTCAAAACCCAGTCGCGACCATGAAAAGTGACCATGAAATCACGATAAGATAGACAGAACAGAATAACCAACCTATAACCGTTCATTGTTATTAAATTTACCCCACATACACGACGCACCACACAGCCTTACCTCTCTCTACTCCAACC

>probe_AGO1

CTAGTGCGATTGCCATTGAGTGGCTCAGGGACACTCTGCGCTGGAGGGGGTCCTAGACAATAGGAAACCCAAGTGTTGTTCGCAGCTCGCTCTATCACATTGTGGTTCTTGCGCCAGCCCGCTTAGGAACGGTGCGTGATCGAAACAATCAACTTCGACCCAAGTTTATCATCGTGTTTATTTTATCGGGAAAGCATTCATCAACATGTCTCCAACCCCGCCATCCACGACCTCGTCAAACGCTAGCGCATTTTCGCCCGAAGGACAGTACAGGGTCATCCGAAAGAGAAATCGGGTGCCACTTTCCTGTGGCCCCTGCCGACATAGGAAGTAAGATTCGTACCCTCTCTCTGAGACCTTTTCGAGCAATTTCTGACAGTTCGTGACCAAGACTCAAATGTAACCGTGCTCACCCATGTGAAAACTGCGTGAAACGAGGAGATGCTGCCTCGTGTAC

>probe_AGO2

CTGTCCATCCATGTGGGAAGCCGACATTCATTGCGTTGTCTTCTATTACCCGAGTCAAGTGTAAACGCTTCACAGGATGGCGTTCTCCCTATATAAGAAAACCCTCTCTTCCTCCCTATGGAACTTACTCTCCTTCTCTCGCCTTTCTCATATCTGTGTGAACTTGCTGCCCTGCTTTGTTTCTTTTATATTGCCACTCTCCTGGTCTCATACCAGTAACCTTTATTTTCCCTTCTACCATGGAGGAATTCGAAGTCGTCAATAAACTTACTGGCGGTCTCCGCCACCTCCGAGTTCGTGAAGCTGACGAAGTCGTCAAGTATTCTGTTCCAGTCGAAACTGTGCCTCGTCCGGGCTTCAACACCACGGGCAAAGAAGTGGACATTTCTCTGAACGCCTATCCGATCACCAAGTTCCCTAGTAGGAACGTTTACCAGTACGATGTGAGTGTCTCCGGCAGCCATAGTATCTTGTTTGACTCAGCC

>probe_RDRP1

GTGACGATATAGCTCAAGTCTTGCATGACTGGATATTGATAAAGGTGAACGGCACGTTTTGAGATCGTAAACATGTAGACTGGCTTTCAATCGATATCATACACATTGGTAGTGACTTACATGGACTGGTTGGCTAACCGAGAAGTCACGAGATGTACTAAGCAAACAACCGCTTTCTAGTAGCACTGACTGCTTGAAATTCATATTTCTATACTATGATACTGATCATGGATTGAGAATTTATGTCTTCAAGGTTTAGGTCATAAGGAATATGAGAAACTATCCTGGTATTCCACAGAGTGATTACATTTGCTAAACGTAGAAAATAATAAATATCATATCATGGGCTGCTAATACCGCTATAGGCTTTACCTACATACTCAGGGCTGTATGTACCAGAGACCATTAGTCCAACTTATCAGCATAGGCCGTGCTGCCAGATAAAAACATATTCAAACTATCGATGCCTGACCCTGTATCTTGGTAGCCAGACGTTTTTCATAAGTATCACATAGTAAAACAATTCTTCGGATCAGCCTTGTCGCGATGATCTAACTCGGCTAACAGTGCACAGGCCGCGACAACTCGGAAGCTCTTAAGCTCACCGTATACCTCCTTGCG

>probe_RDRP2

GGACTAGCGATCTGAGGTTCTTCACGATTCAAGTCAAGTCCAATGTTGATGACTGACGTTCGACCGTTGGGAAGCTTGATGGTGTATAATCCCTTCCTCCAAAAATCTGTTTTAGGAGGTGGTCTGTCGAGATGTGAGAACAATTAGTAGTTGTATAGTGAGAGTTCAGTGAGTCTTACTTGAAGCGGATTTTCCCTCTAGAGTCTCTGTTGCCATGAGAGTCCTCGAATAGATCAATAGAAGAGACATAGCCCTCGCTCGAGAATGTTTGCCAAAGGATACGTGTGTTAATTTCCCTCGGAAGGTTGAATAGGTTCACAGCTACAGTATCCCAGAATGTCCATGGAGCCAGCAATAACTGGGCCGGCTGTTGTTGCAGCCGAGATGAATTCTGAGGCTGACGTGAGGTACCTTGACCGGGGCGGCCAAAGTCTCTCGAGCGGTTACCACGACGATTATGTGACATGGTGGTCAATGATCGCGATGATCGACGGTTGAAAGCGGAAGATGAGGTGGAAGAGGATGAAAGAGAGGGAGAATGTGAACGTGAATGAAGGAAAGGGTCAGTTGTGTTGTGAAGGCAGCTCTCACTAGTGGTAAGGGCACGATGGGGACTGACACCAGTCTGGTATG

>probe_RDRP3

GCATCAAGATCATGCTACTCCTTTGGATGTAATTACTCCAATTCTCTCGACTTTTATTGAGCTATCTTTCCTGGTAGGCGACCCATGCCCCCAACGTAAGGGTTTGCGTGTGAACCACATTTCCAAGGCTTCATGAGGATCCTGCACCCTCGTAGATACTACAGGTTCCGTTAATCAAAAAATGCCTGCAAGTTCTCGAGGTTCGAAGACTTGCGGACAAAGATCTCCAAACTGTCCGGTGTCATGGTAGCAATGCGACTCTTCCGCCGCGCCTCTCTATCAATCAACAGGAGCACAAATTGCCCTGAAGCTGACGAACCAGCCGATACATTTTTCTTACTCCGTCTTTCCCATTGCAGGATCATTCTGTCTGGATCGGAGCTGTCTCTGTTTTCGTCGTTGATGATGAATCGGTAATGTACAATTCATTGACAGTCGCATAAACATCACCCGTCCTCAAGCTGACGCTTTGAGGAAAGTCGCGAGTCATTGATTTGCGAAATATCGGTTGCCATATTCCAGTACCAGTATTGGTCGGTGATTCTGAGCGAGATGTTGGATTTGGAGAAGCTTCTGGATAAGTATAACATGTTGTGACTAGCCCTTTCGTAATATCATGAGATGGTCCTGCG
