# Supplementary figures and images for: RNA interference of Aspergillus flavus in response to Aspergillus flavus partitivirus 1 infection
Source: Front Microbiol. 2023 Nov 14;14:1252294. doi: 10.3389/fmicb.2023.1252294 (PMC10682719; doi:10.3389/fmicb.2023.1252294)

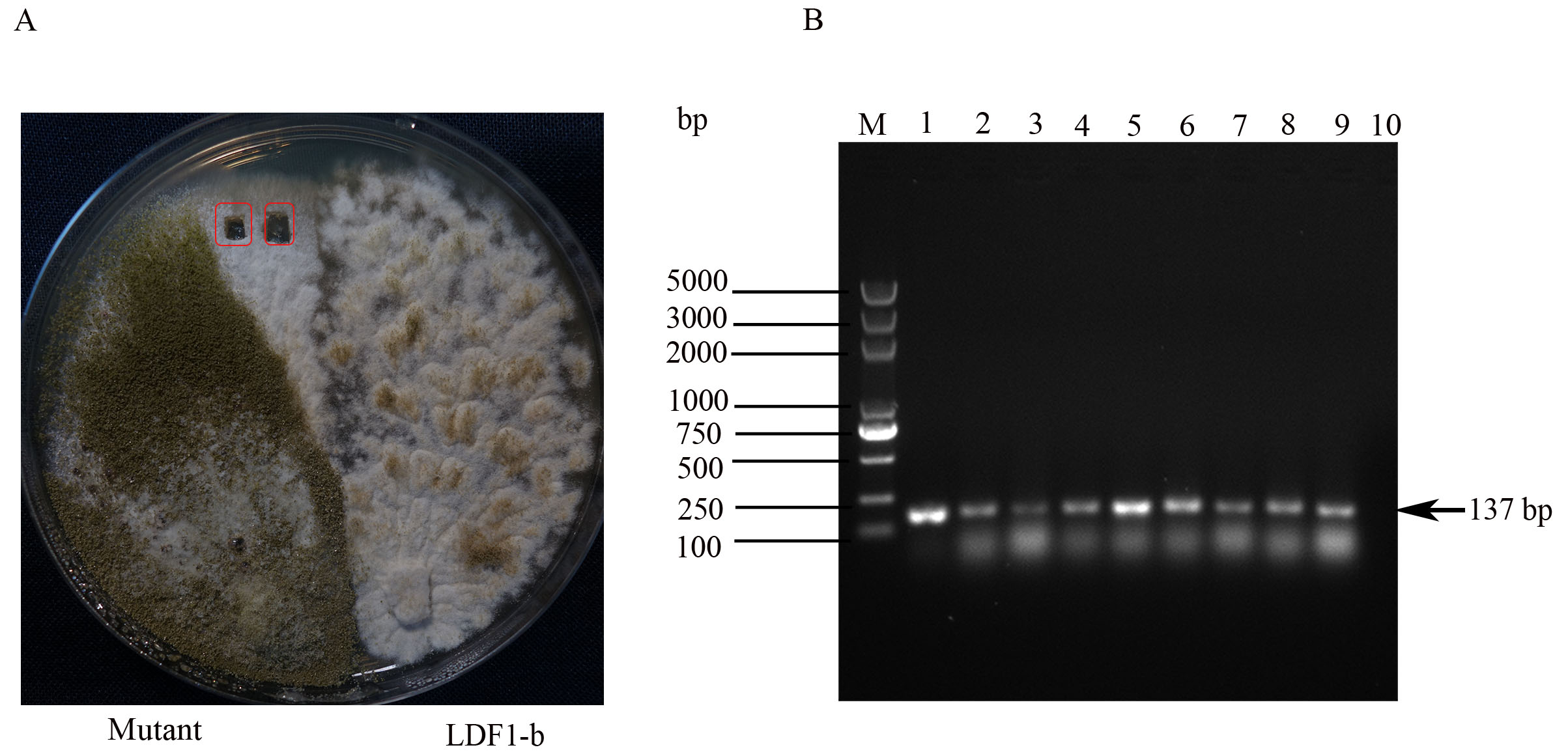

Supplement: Supplementary file 4 [file Image_1.JPEG]

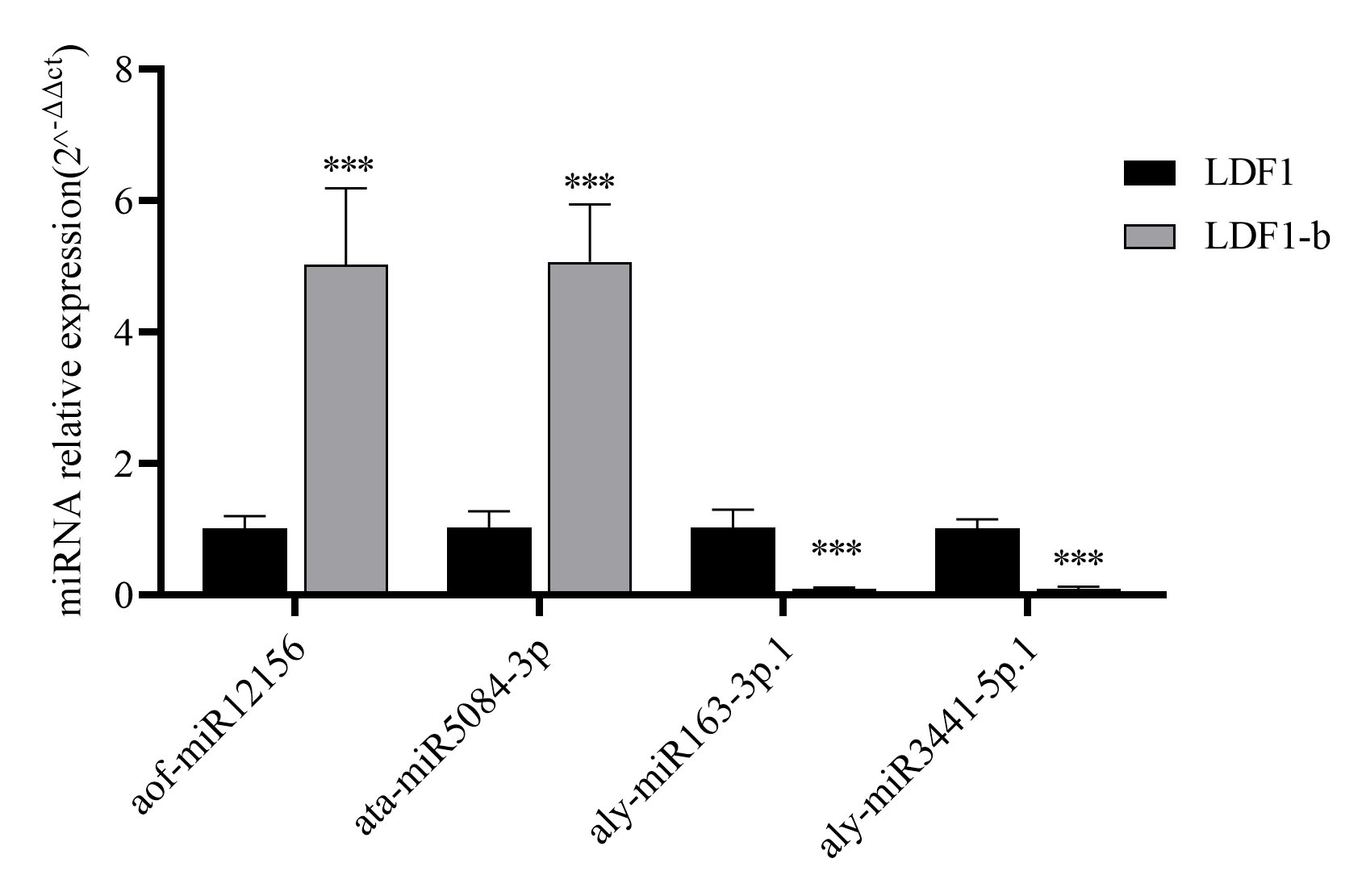

Supplement: Supplementary file 5 [file Image_2.JPEG]
